# Assessing the impact of the Royal Canadian Mounted Police (RCMP) protocol and Emotional Resilience Skills Training (ERST) among diverse public safety personnel

**DOI:** 10.1186/s40359-022-00989-0

**Published:** 2022-12-09

**Authors:** R. Nicholas Carleton, Michelle McCarron, Gregory P. Krätzig, Shannon Sauer-Zavala, J. Patrick Neary, Lisa M. Lix, Amber J. Fletcher, Ronald D. Camp, Robyn E. Shields, Laleh Jamshidi, Jolan Nisbet, Kirby Q. Maguire, Renée S. MacPhee, Tracie O. Afifi, Nicholas A. Jones, Ronald R. Martin, Jitender Sareen, Alain Brunet, Shadi Beshai, Gregory S. Anderson, Heidi Cramm, Joy C. MacDermid, Rosemary Ricciardelli, Rasheda Rabbani, Taylor A. Teckchandani, Gordon J. G. Asmundson

**Affiliations:** 1 Anxiety and Illness Behaviours Laboratory, Department of Psychology, University of Regina, Regina, SK S4S 0A2 Canada; 2 Research Department, Saskatchewan Health Authority, Regina, SK S4S 0A5 Canada; 3 Department of Psychology, University of Regina, Regina, SK S4S 0A2 Canada; 4 Treatment Innovation for Psychological Services Research Program, Department of Psychology, University of Kentucky, Lexington, KY 40506 USA; 5 Faculty of Kinesiology and Health Studies, University of Regina, Regina, SK S4S 0A2 Canada; 6 Department of Community Health Sciences, University of Manitoba, Winnipeg, MB R3E 0W3 Canada; 7 Department of Sociology and Social Studies, University of Regina, Regina, SK S4S 0A2 Canada; 8 Faculty of Business and Economics, University of Northern British Columbia, Prince George, BC V2N 4Z9 Canada; 9 University of Regina, Regina, SK S4S 0A2 Canada; 10 Canadian Institute for Public Safety Research and Treatment, University of Regina, Regina, SK S4S 0A2 Canada; 11 Department of Kinesiology and Physical Education, Wilfrid Laurier University, Waterloo, ON N2L 3C5 Canada; 12 Department of Community Health Sciences, University of Manitoba, Winnipeg, MB R3E 0W5 Canada; 13 Department of Justice Studies, University of Regina, Regina, SK S4S 0A2 Canada; 14 Faculty of Education, University of Regina, Regina, SK S4S 0A2 Canada; 15 Department of Psychiatry, Department of Community Health Sciences, University of Manitoba, Winnipeg, MB R3E 0W5 Canada; 16 McGill’s Psychiatry Department and Douglas Institute Research Center, 6875 Lasalle Boulevard, Verdun, QC H4H 1R3 Canada; 17 Faculty of Science, Thompson Rivers University, Kamloops, BC V2C 0C8 Canada; 18 School of Rehabilitation Therapy, Faculty of Health Sciences, Queen’s University, Kingston, ON K7L 3N6 Canada; 19 School of Physiotherapy, Western University, London, ON N6A 3K7 Canada; 20 School of Maritime Studies, Fisheries and Marine Institute, Memorial University of Newfoundland, St. John’s, NL A1C 5R3 Canada; 21 George & Fay Yee Centre for Healthcare Innovation, Community Health Sciences, Max Rady College of Medicine, Rady Faculty of Health Sciences, University of Manitoba, Winnipeg, Canada

**Keywords:** PTSD, Posttraumatic stress injuries, Potentially psychologically traumatic events, Longitudinal, Risk, Resiliency, Transdiagnostic, Unified protocol, Emotional Resilience Skills Training

## Abstract

**Background:**

Public safety personnel (PSP; e.g., border services personnel, correctional workers, firefighters, paramedics, police, public safety communicators) are frequently exposed to potentially psychologically traumatic events. Such events contribute to substantial and growing challenges from posttraumatic stress injuries (PTSIs), including but not limited to posttraumatic stress disorder.

**Methods:**

The current protocol paper describes the PSP PTSI Study (i.e., design, measures, materials, hypotheses, planned analyses, expected implications, and limitations), which was originally designed to evaluate an evidence-informed, proactive system of mental health assessment and training among Royal Canadian Mounted Police for delivery among diverse PSP (i.e., firefighters, municipal police, paramedics, public safety communicators). Specifically, the PSP PTSI Study will: (1) adapt, implement, and assess the impact of a system for ongoing (i.e., annual, monthly, daily) evidence-based assessments; (2) evaluate associations between demographic variables and PTSI; (3) longitudinally assess individual differences associated with PTSI; and, (4) assess the impact of providing diverse PSP with a tailored version of the Emotional Resilience Skills Training originally developed for the Royal Canadian Mounted Police in mitigating PTSIs based on the Unified Protocol for the Transdiagnostic Treatment of Emotional Disorders. Participants are assessed pre- and post-training, and then at a follow-up 1-year after training. The assessments include clinical interviews, self-report surveys including brief daily and monthly assessments, and daily biometric data. The current protocol paper also describes participant recruitment and developments to date.

**Discussion:**

The PSP PTSI Study is an opportunity to implement, test, and improve a set of evidence-based tools and training as part of an evidence-informed solution to protect PSP mental health. The current protocol paper provides details to inform and support translation of the PSP PTSI Study results as well as informing and supporting replication efforts by other researchers.

***Trial registration*:**

Hypotheses Registration: aspredicted.org, #90136. Registered 7 March 2022—Prospectively registered. Trial registration: ClinicalTrials.gov, NCT05530642. Registered 1 September 2022—Retrospectively registered. The subsequent PSP PTSI Study results are expected to benefit the mental health of all participants and, ultimately, all PSP.

**Supplementary Information:**

The online version contains supplementary material available at 10.1186/s40359-022-00989-0.

## Public significance statements/highlights

- Research on how to mitigate posttraumatic stress injuries (PTSIs) among public safety personnel (PSP) who are exposed to potentially psychologically traumatic events is limited.
- The PSP PTSI Study has been designed to develop, implement, and assess the impact of tools and skills designed to proactively mitigate PTSIs.
- PSP recruited into the study to receive the augmented training are assessed before and after training, and again 1 year later.
- The PSP PTSI Study results are expected to benefit the mental health of all participants and other PSP by informing strategies to mitigate PTSI.

**Supplementary Information:**

The online version contains supplementary material available at 10.1186/s40359-022-00989-0.

## Background

Public safety personnel (PSP; [1]) include diverse professionals (e.g., border services personnel, correctional workers, firefighters, operational and intelligence personnel, paramedics, police, public safety communicators, search and rescue personnel). PSP report extremely frequent and severe exposures to potentially psychologically traumatic events (PPTEs), often reporting more than 11 exposures to each different type of PPTE [2]. Nearly half (i.e., 44.5%) of PSP screen positive for one or more posttraumatic stress injuries (PTSI; e.g., major depressive disorder, panic disorder) at any given time [3]. Many PSP report suicidal behaviours during the past-year (i.e., ideation [10.1%], planning [4.1%], attempts [0.3%]) or during their lifetimes (i.e., ideation [27.8%], planning [13.3%], attempts [4.6%]) [4].

There are increasing commitments and efforts to provide evidence-based supports for PSP mental health [5, 6], but the available evidence regarding what will render the strongest effects remains sparse [7–11]. There are dozens of programs purported to support PSP mental health, most focusing on increasing knowledge, reducing stigma, and increasing help-seeking behaviors, but almost none have peer-reviewed research regarding effectiveness [7, 12]. Most of the peer-reviewed studies use cross-sectional data, short follow-up periods, and assess very small subsets of variables posited as important for mental health [7, 12, 13]. The limited available evidence suggests the extant programs produce benefits that are small, time-limited, and highly variable [8, 9, 11, 14, 15], due in part to low delivery fidelity [16] and insufficient mechanisms of action for mitigating PTSIs [7, 11, 12]. A particularly robust trial that compared psychoeducation to resilience training among PSP demonstrated no statistically significant differences between groups or over time in improvements on modifiable individual differences previously posited as PTSI risk factors [17].

Potentially modifiable risk factors for PTSI include elements of some personality traits (e.g., extraversion, conscientiousness, neuroticism, world view) [18, 19], hope [20], distress tolerance [21], optimism [22], interpersonal supports [23], and positive life activities (e.g., exercise) [24]. There are also individual differences on other psychological variables, such as anxiety sensitivity [25], fear of negative evaluation [26], illness/injury sensitivity [27], pain-related anxiety/fear [28], intolerance of uncertainty [29], rumination [30], maladaptive self-appraisal [31], dissociation [32], and anger [33], as well as differences in physiological variables, such as autonomic nervous system dysregulation [34–37]. Additional details regarding individual differences of interest for the PSP PTSI Study and the Royal Canadian Mounted Police (RCMP) Study are available in the Additional file 1. Environmental factors—such as PPTE and other stressors (e.g., adverse childhood experiences, difficult socioeconomic status) [38]—can also serve as risk factors for PTSIs. Other risk factors (e.g., family history of psychopathology [39] and aversive avoidant reactions to emotions) appear associated with PTSI development [25, 29] and may be managed by learning to accept and manage emotions instead of using avoidant coping strategies (e.g., alcohol and drug use, avoiding reminders of the event) [40–43].

The Unified Protocol for the Transdiagnostic Treatment of Emotional Disorders (UP) [41, 42] is an evidence-based cognitive-behavioral intervention designed to cultivate constructive approach-oriented emotional engagement. The UP is supported by considerable research evidence demonstrating transdiagnostic therapeutic effectiveness across several delivery formats (e.g., individual, group, self) [40, 44–48]. There is preliminary support from a randomized trial that the UP may be an effective proactive intervention to mitigate PTSI based on results from participants with elevated nonclinical symptoms of depression and anxiety reporting statistically significant improvements from baseline to 1-month follow-up [49]. The participants described the proactive UP training as highly satisfying and acceptable, and reported using the new skills “some” to “most” of the time, even at 1-month follow-up. The available evidence supports the UP as a promising proactive intervention that can be efficiently and effectively delivered to protect PSP mental health [13, 46, 50–54] and enhance job satisfaction [55].

In 2019, the RCMP undertook a large, multi-faceted, and multi-modal research study (i.e., “the RCMP Study”; www.rcmpstudy.ca) with several over-arching objectives [56]: (1) to develop, implement, and assess the impact of a system for ongoing (i.e., daily, monthly, annual) evidence-based biopsychosocial assessments (i.e., biometrics, clinical interviews, self-reported symptoms, social experiences); (2) to adapt the UP [40, 41], creating the Emotional Resilience Training (ERST); (3) to augment the RCMP Cadet Training Program [52, 57, 58] with the ERST to proactively mitigate PTSI; (4) to assess the impact of the tailored training on individual differences over time (i.e., pre-training, post-training, follow-up) and relative to the standard training; (5) to evaluate associations between demographic variables and PTSI symptoms; and, (6) to longitudinally assess individual differences associated with PTSI symptoms. The RCMP Study is a much-needed large-scale evaluation of a proactive, pervasive, and perpetual mental health assessment and training protocol designed to mitigate PTSI [12]. The RCMP Study required the development of several assessment protocols, as well as adapting the UP into the 13-week ERST and integrating the training into the RCMP Depot Division Cadet Training Program.

In 2020, the Canadian Institutes for Health Research awarded funding for “An Augmented Training Program for Preventing Post-Traumatic Stress Injuries Among Diverse Public Safety Personnel” (i.e., the PSP PTSI Study). The PSP PTSI Study was designed to adapt the RCMP Study protocols and ERST training [56] to accommodate a diverse range of participating PSP. The current protocol paper describes the PSP PTSI Study(i.e., design, measures, materials, hypotheses, planned analyses, expected implications, limitations), which is part of the concerted efforts being made by the Government of Canada, the Canadian Institutes for Health Research, and the Canadian Institute for Public Safety Research and Treatment to develop and implement evidence-based solutions to support PSP mental health [6].

The over-arching objectives for the PSP PTSI Study are to: (1) adapt, implement, and assess the impact of a system for ongoing (i.e., pre-training, post-training, follow-up, daily, monthly) evidence-based assessments of environmental factors and individual differences (i.e., measurements of biometrics, mental health, social experiences); (2) evaluate associations between demographic variables and symptoms of PTSI; (3) longitudinally assess environmental factors and individual differences associated with PTSI; (4) adapt the RCMP Study ERST to accommodate a diverse range of PSP; (5) implement the ERST with a diverse sample of PSP; (6) assess participant reactions to the ERST; and, (7) assess the impact of the ERST on individual differences and symptoms of PTSI over time. The current protocol paper: (1) details the PSP PTSI Study design, protocols, measures, materials, and hypotheses; and, (2) describes participant recruitment and study progress to date.

## Methods

### Study design

The PSP PTSI Study uses a longitudinal prospective sequential experimental cohort design [59–61] that engages each participant for approximately 16 months. Several practical constraints prohibited a randomized controlled trial, including resource limitations and probable interactions between PSP during and after training. Nevertheless, evidence from meta-analyses suggests against statistically significant differences between observational studies and randomized controlled trials, irrespective of specific design [62, 63], and the RCMP Study design is expected to generate results relative to a control group [56].

### ERST adaptation

A focus group was conducted with PSP PTSI Study stakeholders prior to the first training session to inform adaptation and tailoring of the ERST, shifting from RCMP-focused content to engage a broader PSP audience. The work included identification of gender- or sex-related considerations. The focus group met three times over 3 days and was led by one of the co-authors (M.M.). The focus group participants included a firefighter, two paramedics, a police officer, and a public safety communicator. The stakeholder feedback was transcribed and analyzed to identify necessary adaptations, all of which were completed initially by another co-author (R.E.S.), reviewed by the stakeholders, and the reviewed by another co-author (S.S-V.) to check for fidelity with the UP. The updated content was reviewed by the PSP ERST trainers who made additional revisions, all of which were reviewed by the same co-author team (M.M., R.E.S., S.S-V.) before finalizing the materials.

### Ethics approval

The University of Regina Ethics Board provided initial approval on May 28, 2021 (File #2020-226) and renewed on May 28, 2022. The trial is relatively short-term, relatively low risk, focused on symptom relief, and is being independently overseen by the University of Regina Research Ethics Board; as such, a dedicated data monitoring committee was created.

### Participant information

#### Inclusion/exclusion criteria

Participants include 200 currently serving PSP recruited from CanOps, Regina Fire & Protective Services, Regina Police Service, Saskatoon Police Service, Regina Emergency Medical Services, Ottawa Emergency Medical Services; accordingly, potential participants were Canadian citizens or permanent residents, 18 years or older, who fluently read, write, and speak either English or French [64]. Participants were recruited from four PSP sectors: firefighters, municipal police officers, paramedics, and public safety communicators. Inclusion criteria included employment in their sector for a minimum of 3 years, and ability to access a computer with internet service. Exclusion criteria included high risk of suicide or previous suicide attempt/hospitalization within the prior year, currently experiencing psychosis, mania, or impairing drug or alcohol addictions, current or ongoing performance management concerns, and any history of advocating against mental health care.

#### Sample size requirements and power analyses

Power analyses for a cluster randomized trial were performed using SAS version 9.3 (SAS Institute Inc., Cary, NC, US) and RMASS. Based on these analyses, a sample of 40 participants per sector at Time 2 will provide adequate power (i.e., > 80%) [65, 66]. Power analyses assumptions were: (1) clustering of participants into sectors; (2) repeated measures in participants over time (i.e., clustering within participants); and, (3) attrition (i.e., loss to follow-up).

#### Longitudinal and cross-sectional continuous outcomes (e.g., PTSI symptoms)

Power associated with the sample size was calculated to determine the statistically significant effect size (the standardized difference in means). Repeated measures on ANOVA (with statistical software G*Power) used for the calculation, the calculations were based on statistical power of 80%, setting the sub-sample of 50 participants in each sector (total sample = 200), the nominal statistical significance level at α = 0.05, and the correlation within study participants (i.e., across repeated measurements) assumed to be 0.5. The PSP PTSI Study is well powered for small effect sizes; as such, the effect size should be > 0.10 for overall change from baseline to T2 and T3 (time as a main effect), > 0.11 when calculating differences between sectors (as an interaction effect with time), and > 0.19 when calculating overall differences between sectors (sector as a main effect).

#### Sample size requirements and power analyses for longitudinal and cross-sectional categorical outcomes (e.g., PTSI incidence)

Power associated with the sample size was calculated to determine the significant effect size (odds ratio, likelihood of symptoms). With the exact binomial distributions (with statistical software SAS 9.4; SAS Institute Inc., Cary, NC, US) the calculations were based on statistical power of 80%, setting the sub-sample of 50 participants in each sector (total paired sample = 200), the nominal significance level at α = 0.05, two-sided test and the correlation within study participants (i.e., across repeated measurements) assumed to be 0.5, and the baseline risk symptom proportion = 0.45 [3]. The PSP PTSI Study is well powered for the minimum change from baseline to T2 and T3 with the likelihood of symptom decrease or the odds ratio ≤ 0.66, and by sector the minimum change should have an odds ratio ≤ 0.39, for both longitudinal and cross-sectional analyses.

#### Recruitment and retention

The PSP PTSI Study website (www.saskptsistudy.ca) is publicly available, provides study details, and is actively used by stakeholders; however, the research team reached out to potential participants via email invitations sent by the PSP partner organizations. The email described the study and invited potential participants to reach out to the research team or their PSP partner organizations to volunteer. The PSP PTSI Study team worked with the sector leads to establish the study activities schedule for participants in each PSP sector. Participants were provided with a welcome package via email. The welcome package included an introductory and thank you message from the principal investigator, a copy of the consent form, and instructions for downloading and accessing study applications. Participants were asked to review the consent form and to download and access study applications prior to attending their onboarding session. The onboarding session was conducted over Zoom and provided information about the study rationale, design, requirements, expected outcomes, and potential benefits to the broader PSP community. Potential benefits to individual participants (e.g., monitoring individual mental health, potentiating earlier access to mental health support) were described and participants were given the opportunity to ask questions. The research team then provided a highly detailed and interactive tutorial to facilitate technology set-up (e.g., using the study software and hardware), and another opportunity to ask questions.

Participants can discontinue participation any time. Cumulative attrition from longitudinal studies with PSP who are not able to participate during paid employer hours typically ranges from 3% at 1 year to 43% at 3 years [67–71]. A similar research design with military participants has reported cumulative attrition rates of less than 10% and overall missing data rates of less than 30%, with anecdotal reports that participants appreciate tracking their own activities and symptoms [72, 73]. Some PSP PTSI Study participants were able to participate as part of paid time; as such, attrition rates may be consistent with, or better than, previous research [72, 73], but differences in ability to participate as part of paid time will be assessed as potential covariates.

### Gender and sex

Gender and sex-related factors may affect PSP work experiences and mental health. In a recent large pan-Canadian, pan-PSP study, women in policing and firefighting reported higher prevalence rates of posttraumatic stress disorder (PTSD) and other mental health disorders; however, the differences were not statistically significant for correctional workers or paramedics [3]. Women continue to be under-represented across most PSP sectors in Canada [74]. Existing research has documented concerns about hyper-masculinity and sexual harassment affecting women in policing [75–78], and recent high-profile legal cases have highlighted ongoing sexual harassment and assault in Canadian policing [79] and corrections [80], and amongst American paramedics [81]. Gendered ideologies linking physical strength with competency may further sideline female officers [82]. Women PSP experience such work-related stressors in combination with other gendered stressors in everyday life. For example, women in Canada continue to perform disproportionate amounts of domestic and caregiving work [83], which creates specific challenges for women accessing parental leave and balancing work with caring responsibilities [25, 84].

The researchers will convene eight subsequent sector-specific focus groups after T2 (i.e., immediately after program completion), separated by position (i.e., frontline, leader) and sector, to support evaluating the training content, delivery, and impact. The eight focus groups will also identify any differential experiences of the training based on PSP sector or other variables, including gender, identify causal factors, and propose solutions for future training where appropriate. The eight focus groups will include both men and women; however, two additional separate focus groups will be convened, one for women across all PSP sectors and one for men across all PSP sectors, to identify gender dimensions of PSP experiences. The result will be a total of 11 focus groups for the current study (i.e., *n* = 1 initial; *n* = 8 after T2; *n* = 2 after T2).

Demographic survey questions were asked about both gender and sex to avoid conflation [85]. To facilitate inclusion of diverse gender identities, the gender question included options for “transgender,” “non-binary,” and “Two Spirit” in addition to “man” and “woman”. Gender and sex questions also provided an open, write-in option. The PSP PTSI Study will therefore support sex- and gender-disaggregated analyses across various topics (see Additional file 1: Tables). Quantitative results will be disaggregated by both gender and sex, as well as sector, rank, and other demographic intersections (e.g., age) to assess for potentially important nuances. Qualitative analyses will inductively explore any differences in experience due to gender or other diversity factors (e.g., ethnicity, age) in open-ended survey responses and focus group data. Sex and gender analyses will be designed and led by Fletcher, who is a gender sociologist and the identified project gender champion as required by the funding agency.

### Data collection time frame

Participants will each be assessed for a period of at least 16 months to identify pre-training, post-training, and follow-up results associated with ERST. Study assessments occur in four main categories (see Tools and Measures for details): Full Assessments, Monthly Assessments, Daily Assessments, and Biometric Assessments. The data collection time-period broadly uses three milestones: pre-training (i.e., T1), post-training (i.e., T2; ~ 16 weeks after training commences), and a follow-up 1 year after T2 (i.e., T3). Each milestone involves a Full Assessment (i.e., FA1 to FA3). Data collection concludes at FA3. Participants complete their first Monthly Assessment (i.e., MA1) approximately 4 weeks after completing FA1 and do not complete a Monthly Assessment concordant with a Full Assessment (i.e., maximum number of Monthly Assessments per participant is 15). Participants can complete their first Daily Assessment (i.e., DA1) on the same day as FA1 (i.e., maximum number of Daily Assessments per participant ~ 540).

### ERST adaptation and training details

The ERST is a 13-week program developed as an adaptation of the UP [40, 41]. The UP, and therein the ERST, focuses on skill development to offset individual differences that lead to emotional disorders by facilitating an approach-oriented stance toward emotions and reducing reliance on avoidant coping strategies. The ERST frames emotional experiences as natural responses to threat, rather than pathological occurrences to avoid; as such, the ERST is well-suited for mitigating health challenges and the skills may also help PSP to support persons in distress, including other PSP and the community members they all serve.

The RCMP ERST training materials include an instructor guide, didactic PowerPoints, and a trainee workbook. The RCMP ERST [56] was adapted for use by a broader range of PSP based on feedback from our PSP knowledge users (i.e., C. Ward, K. Digney, K. Luciak, D. Milo, M. Pritchard) and a focus group of PSP stakeholders (i.e., K. Breitkreuz, T. Frei, C. Gottselig, K. Luciak, M. Slater). The focus group was conducted and recorded via Zoom over three sessions. Participants were asked to review the materials prior to the sessions, and then the focus group leader asked a series of questions (e.g., “Bearing this in mind for your particular sector, how realistic do you feel this example is? Is this something that people can relate to?”) to elicit feedback and suggestions from participants. The session transcriptions were used to adapt the materials for use by a broader range of PSP. For example, scenarios related to RCMP cadet training were replaced with yearly continuing education scenarios designed to be relatable for a broad range of PSP. Participants were asked to do a final review of all materials after the initial adaptations were completed. The materials were then revised if needed and sent to the research team ERST leader (Sauer-Zavala, a co-developer of the UP) to ensure the adaptations complied with UP fidelity requirements.

The ERST training is designed as a “train the trainer” model. Sauer-Zavala personally trained a group of PSP trainers (i.e., C. Brandauer, J. Earl, T. Frei, A. Gabriel, K. Gifford, J. Hedlin, A. Hemsworth, L. Kolybaba, L. Marshall, B. Onyskevitch, C. Wolbaum) from each of the PSP pilot sectors (i.e., CanOps, Regina Fire & Protective Services, Regina Police Service, Saskatoon Police Service, Regina Emergency Medical Services, Ottawa Emergency Medical Services) during a week-long interactive workshop. The trainers continue to have access to Sauer-Zavala for optional follow-up consultation and support related to delivery of the ERST training for questions or to address any issues that arise during training. Having consultation and support available for the trainers should help to offset concerns raised about ensuring training fidelity subsequent to other mental health programs [12, 16]. Participants will have ongoing access to ERST to support skill retention after training is completed, which should help to offset previous indications of problems with skill development for mental health programs [7, 8, 12, 14, 15].

### Tools and measures

#### Tools—moodle and qualtrics

All communications between the research team and participants, administration of surveys, feedback for participants (including their clinical assessment reports), and the ERST materials are coordinated through a tailored and dedicated instance of Moodle (i.e., the Portal) paired with a software application (i.e., the App) downloadable onto compliant smart phones. Surveys are administered in English or French through a secure Qualtrics account. Clinical Interviews are supported by electronic administration of the Mini-International Neuropsychiatric Interview (M.I.N.I.) [86–88].

#### Full assessments—full surveys

Details of the Full Surveys (e.g., questionnaire titles, details, psychometric information, references) are in the Additional file 1: Psychometrics and References for Self-Report Measures. The initial Full Survey assesses demographics not expected to change, or which are unlikely to change, during the study (i.e., sex, age at T1, height, ethnicity, gender, sexual orientation, adverse childhood experiences), as well as pre-recruitment demographics (i.e., education, employment, language(s) spoken, religion, work history, rank, work hours, living location, and mental health history—all recorded prior to recruitment). All Full Surveys assess demographics that could plausibly change during the study (i.e., physical health conditions, socio-economic status, marital status, and household composition). Average completion time for each Full Survey is 120 min. Participants can access their Full Survey results in a dedicated report containing context and academic references through the Portal.

All Full Surveys also assess for: (1) symptoms of PTSIs including generalized anxiety disorder, major depressive disorder, panic disorder, PTSD, and social anxiety disorder, as well as PTSI correlates (e.g., substance use, chronic pain, insomnia, marital satisfaction, stress, diagnoses since the last assessment); (2) environmental factors and individual differences associated with PTSI: (a) PPTE exposures; (b) personality; (c) anxiety sensitivity, fear of negative evaluation, illness/injury sensitivity, intolerance of uncertainty, pain-related anxiety, resilience, moral injury, anger, mindfulness, beliefs about emotions, experiential avoidance, emotion regulation, mindfulness skills; (d) mental health care knowledge, access, and use; (e) occupational stressors, work fulfillment, institutional betrayal, work-related discrimination, work-related sexual harassment and assault, stigma, family stressors, posttraumatic growth, social support, self-care, overall well-being; and, (3) ERST retention and use. Responses consistent with one or more mental disorders are flagged for participants and include recommendations for accessing additional mental health supports.

#### Full assessments—clinical interviews

Each Full Assessment includes a semi-structured M.I.N.I. Clinical Interview [86–88] conducted by a registered clinical psychologist or supervised trainees. The M.I.N.I. provides a standardized diagnostic approach consistent with DSM-5 criteria [89]. The interviewer reviews the Full Survey results prior to M.I.N.I. administration in either English or French, per participant preference. The published M.I.N.I. inter-rater reliability exceeds 75% [86, 87]. Average completion for each clinical interview is 45 min. Participants receive verbal (from the interviewer) and written (through the Portal) summaries of the results from their Full Assessment and are advised of responses consistent with one or more mental disorders and referred for mental health support as indicated; however, a formal diagnosis is not provided because that is beyond the study scope.

#### Monthly assessments

Details of the Monthly Assessments (e.g., questionnaire titles, details, psychometric information, references) are in the Additional file 1: Psychometrics and References for Self-Report Measures. The maximum number of Monthly Assessment questions is 293, determined by participant responses to header questions. Average completion time for each Monthly Survey is 32 min. All Monthly Surveys also assess for: (1) symptoms of PTSI including generalized anxiety disorder, major depressive disorder, panic disorder, PTSD, and social anxiety disorder, as well as PTSI correlates (e.g., substance use, chronic pain, insomnia); (2) individual differences associated with PTSI: (a) PPTE exposures; (c) resilience; (d) mental health care knowledge, access, and use; (e) occupational stressors, social support, and self-care; and (3) ERST retention and use. Participants receive written (through the Portal) summaries of their Monthly Assessment including timeline charts for participants to monitor fluctuations, facilitating participation healthy habits [90–92]. Responses consistent with one or more mental disorders are flagged for participants and include recommendations for accessing additional mental health supports.

#### Daily assessments

The Daily Assessments are very brief self-report questionnaires that allow participants to reflect and report on the past 24 h for (1) Mood, Attitude, and Performance; (2) Physical Wellness; (3) Emotional State; (4) Work Hours; (5) Sleep Hours; (6) Sleep Quality; (7) Eating; (8) Physical Activity; (9) Social Activity; (10) Substance Use and Gambling; and (11) ERST use. Mood, Attitude and Performance, and Physical Wellness are rated on 100-point visual analogue scales with anchors of ill (0–25), injured (26–50), reacting (51–75), and healthy (76–100). A 24-point rating scale is used for Emotional State, Work Hours, and Sleep Hours. Sleep Quality, Eating, Physical Activity, Social Activity, and Substance Use is reported dichotomously (i.e., yes/no), with discretionary options for participants to record details. The Daily Assessments also allow participants to log PPTE or other significant emotional events, creating a record of exposures to stressors. Average completion time for each Daily Survey is ~ 1 min. Completing the Daily Assessments supports regular self-reflection and provides participants with graphical feedback to facilitate healthy habits [90–92].

#### Biometric assessments

The LLA Recordis™ cardiac sensor device (LLA Technologies Inc., Langley, BC, Canada) is used to collect timing data that are similar in quality to M-mode echocardiography timing events (i.e., systole, diastole, isovolumic contraction and relaxation periods, rapid ejection), as well as twist forces of the ventricle (i.e., surrogate for contractility), heart rate variability (i.e., low frequency, high frequency, low frequency/high frequency ratio, square root of the mean of the squares of the successive differences between adjacent NNs), and Myocardial Performance Index variant [93]. The Recordis™ is applied to the sternum base (~ 1 cm above the xiphoid process) with a single electrocardiography electrode or heart belt strap, and uses a smartphone application providing immediate and ongoing user feedback. The current protocol requires a 1-min recording upon waking, with data downloaded for off-line processing and analyses. Briefly, previous research using echocardiography, the “gold standard” for cardiac function analysis, has demonstrated that individuals with PTSD have reduced diastolic function [94]. The LLA Recordis device can be used to record cardiac timing [95] events synonymous with echocardiography (e.g., diastole, systole, isovolumic relaxation, and contraction time).

### Data management and confidentiality

Data transfers from participant devices to secured research servers in Canada are protected using Transport Layer Security. The PSP PTSI Study also employs a PKI Class 3 SSL Certificate, with a 2048-bit digital signature and 256-bit encryption. Data stored on the servers are automatically encrypted using server-side Advanced Encryption Standard–256 encryption before being saved to disk and decrypted before data are downloaded. The data are stored separately from the data dictionary and the codes necessary for interpretation.

Study participants are guaranteed confidentially, but not anonymity. Participants provide an email address to manage their interaction and communication with the study. Participant email addresses are then used to log into the study portal and to connect participants with the surveys presented through the study portal. Individual survey results are not available to anyone outside the individual participants who can view their own data and the study team. During the study, the data are accessible only to the study team. Study data are not available to PSP employers.

Participant confidentiality is reviewed at the beginning of each clinical interview, including limitations: (1) imminent risk of harm to self or others; (2) disclosures of child abuse; and, (3) court subpoenas. Participant disclosures of suicidal behaviours during the clinical interview were followed by a SAFE-T Suicide Risk Assessment [96]. Each PSP sector also provided a contact person to call if a participant was at imminent risk of suicide and where necessary 9–11 will be contacted. All participants who reported suicidal thoughts or behaviours were invited to create a safety plan including identifying a specific support plan (e.g., friends, family, significant other, mental health crisis line, 9–11), healthcare professionals and services, and decision planning for when to reach out for help. Participants were also reminded about factors for suicide risk (e.g., alcohol, substance use) and resilience (e.g., social supports, reasons to live).

### Hypotheses

The PSP PTSI Study was registered with ClinicalTrials.gov (i.e., identification code NCT05530642), and the hypotheses were pre-registered.^1^ Hypotheses specific to individual difference variables are provided in Additional file 1: Tables (i.e., PTSI Measures; Primary Individual Differences Associated with Posttraumatic Stress Injuries; Secondary Individual Differences Associated with Posttraumatic Stress Injuries). Overarching PSP PTSI Study hypotheses:

1. Participant mental health disorder prevalence rates at T1, based on Clinical Interviews or screening tools based on self-reported symptoms, will be higher than the general population [3, 97].
2. From T1 to T2, participants will evidence reductions in risk, increases in resiliency, and improvements in mental health, as a function of the ERST [98, 99].
3. Participants will evidence statistically significant predictive relationships between completing assessments and changes to individual differences over time (i.e., inversely with risk, positively with resilience, positively with mental health).
4. Participants will evidence statistically significant sequential predictive relationships for environmental factors or individual differences reported during the *Daily Assessments*, *Monthly Assessments*, and *Full Assessments*.
5. All participants will evidence sustained reductions in risk, increases in resilience, and increases in mental health at T3 relative to T2.
6. Participants will evidence a statistically significant relationship between changes in individual differences over time and engagement with ERST content.
7. Participants will evidence a statistically significant relationship between changes in environmental factors or individual differences over time, frequency of exercise [100], and other self-reported indicators of physical health [101].
8. Relative to men, women will report more difficulties with mental disorder symptoms and occupational stressors.
9. Diastole will be reduced in PSP who report symptoms consistent with one or more PTSI.
10. The biometric data will be statistically significantly and substantively correlated with measures of PTSI.
11. There will be a statistically significant and substantive relationship between PTSI symptom severity and reduced diastolic function.
12. Changes in biological variables (i.e., autonomic nervous system reactivity, heart rate variability, cardiac mechanical changes) will be associated with environmental factors or individual differences.

### Planned analyses

Study data will initially be described using frequencies, means, and standard deviations. Missing data will be described within and across measurement occasions. Multiple imputation will be adopted assuming a missing at random mechanism [102]. Strategies will be adopted to control the familywise error rate (i.e., the probability of at least one Type 1 error in a family of tests) [103], while accounting for Type 2 errors [104]. Analyses will include mixed-effects multiple linear and non-linear regression models including covariates (i.e., sex, age, marital status, education, province of residence) and trend analyses as needed. To test for differences between PSP sectors in the cross-sectional analyses, a mixed-effects model will be used that accounts for clustering within PSP sectors. To test for changes over time, a mixed-effects model will be used that accounts for clustering within PSP sectors as well as within individuals (i.e., repeated measurements for each individual). Recursive Bayes algorithm and ecological momentary analyses will be used to analyze the Daily Assessments and biometric data. Quantitative analyses will be disaggregated for both sex and gender. Qualitative data (survey and focus group) will be coded using conventional qualitative content approaches [105] for inductive identification of themes.

### Knowledge translation

Main results will be shared with the research community via publication in peer-reviewed journals. Results will be of interest to PSP stakeholders (e.g., members, families, leadership), clinicians, policy planners, and governments; as such, knowledge translation efforts will be tailored for different audiences. Technical reports and lay summaries will be available through the study website (www.saskptsistudy.ca), and provided to the PSP knowledge users, the PSP focus groups, and the PSP partner agencies involved in the PSP PTSI Study, as well as provided to the Department of Public Safety and Emergency Preparedness. Results will be communicated to PSP stakeholders through the Canadian Institute for Public Safety Research and Treatment (www.cipsrt-icrtsp.ca) knowledge translation channels, and the University of Regina Communications Department will issue press releases as appropriate.

### Current study status and impacts of COVID-19

Recruitment began after a study launch meeting with the PSP sector leaders on September 22, 2020. COVID-19 substantially impacted the timelines for recruitment and training, as well as increasing the workload for all PSP, which reduced the individual capacity of participants to engage with novel training. In March 2021, a Steering Committee consisting of the PSP sector leads and the research team leads was established and recruitment began in earnest. Steering Committee meetings were held monthly for continued assistance with recruitment. Data collection began on October 20, 2021. T1 data collections for all participants were completed May 23, 2022. The T2 data collections for all participants are expected to conclude by August 19, 2022. The T3 data collections for all participants are expected to conclude by September 16, 2023.

## Discussion

The PSP PTSI Study was designed to develop, implement, and assess the impact of a system for ongoing (i.e., annual, monthly, daily) evidence-based assessments of environmental factors, individual differences in psychological variables, and individual differences in physiological variables (i.e., measurements of biometrics, mental health, social experiences). The PSP PTSI Study is based on the RCMP Study Protocol [56]. The PSP PTSI Study was also designed to prospectively assess for interactions between demographic variables, environmental factors, individual differences, and PTSI symptoms. Data collection is under way and initial results will be reported in subsequent peer reviewed publications. The PSP PTSI Study will test the impact of providing ERST to a diverse sample of experienced PSP. Participants are expected to report significant, substantive, and sustained mental health benefits.

### Expected implications for clinical practice, policy, and research

The PSP PTSI Study was designed to benefit all participants: (1) through evidence-based assessments that facilitate self-monitoring and earlier access to care [90–92]; (2) reductions in mental health stigma through increased discourse [106–109] and social supports [23, 106, 110]; (3) with tangible evidence of organizational commitment to improve evidence-based mental health supports [106, 111, 112]; (4) by creating independent electronic mental health records; and, (5) through shared altruistic engagement in improving mental health for all PSP [113, 114]. The ERST has been tailored by PSP, for PSP, and is being provided by PSP to PSP. The training is based on a transdiagnostic treatment protocol supported by considerable research evidence for therapeutic effectiveness across several delivery formats (e.g., individual, group, self) [40, 44–48] and with preliminary support as a proactive intervention to mitigate PTSI [49]. The ERST dose for the current trial is currently second only to the dose provided through the RCMP Study [56]. The results will provide critical information about the potentially positive responsive and proactive impacts of ERST to help protect PSP mental health.

The PSP PTSI Study was also designed to benefit the PSP stakeholder organizations through evidence-based information for ongoing enhancements to training and assessments, and through implementing and testing a set of tools to pervasively support PSP mental health. The PSP PTSI Study results can also inform assessments, treatments, and programming for diverse PSP, military, veterans, and other persons at risk for PPTE exposures (e.g., nurses) [115, 116]; for example, the PSP PTSI Study results will inform the potential for broad scaling of the RCMP Protocol [56] to all persons at risk for PPTE exposures. The results will also provide critical information regarding logistics of effectively and practically implementing the RCMP Protocol [56] or any other mental health solution for diverse PSP environments. The results will also inform opportunities to improve the protocol and training to better support PSP mental health needs.

### Strengths and limitations

The PSP PTSI Study has several strengths including: (1) longitudinal design elements that can inform causal relationships; (2) prospective design elements that can inform predictive and proactive discussions associated with PPTE; (3) relatively large sample sizes; (4) ongoing multimodal assessments of environmental factors and individual differences, including PPTE exposures, occupational stressors, and social stressors; (5) inter-rater reliability assessments for clinical interviews; and, (6) being an ecologically valid test of the entire RCMP Protocol, including the ERST [56] across diverse PSP.

The PSP PTSI Study also has several necessary limitations. A truly randomized design was impossible because of how PSP interact within their professional environments. The already complex PSP environments were made more so by contemporary challenges with staffing and implementation requirements to protect public safety during the COVID-19 pandemic. Results from the RCMP Study will help address such limitations [56], as will having founded the ERST on the UP content that was based on randomized controlled studies [62, 63]. There is no obfuscation of the intended outcomes from ERST, which will create unknowable influence from expectancy effects. Direct research on such impacts remains nascent [117], but results suggest little or no difference between open and closed label designs when participants are provided with a sufficient study design rationale (e.g., [118]). The assessments are uncommonly detailed, which may produce intermittent participant response fatigue; however, participants were expressly informed about the rationale for the assessment details, which may help sustain morale. The detailed assessments also increase Type I error risks from spurious correlations [119]. The pre-registration of hypotheses, as well as the a priori provision of expected results in the current protocol paper are expected to mitigate Type I error risks and protect against equally problematic Type II error risks [119]. The voluntary nature of participation creates an unknowable influence from self-selection biases. The sample size, analytic plan, relatively short follow-up period, and provision of feedback through an electronic mental health record are designed to offset attrition.

## Supplementary Information

**Additional files 1.** Tables—PTSI_Protocol_Paper_Supplemental.pdf.

## Data Availability

Not applicable.

## Abbreviations

DA: Daily Assessments
ERST: Emotional Resilience Training
FA: Full Assessments
M.I.N.I.: Mini-International Neuropsychiatric Interview
MA: Monthly Assessments
PPTE(s): Potentially psychologically traumatic event(s)
PSP: Public safety personnel
PTSD: Posttraumatic stress disorder
PTSI(s): Posttraumatic stress injury(s)
RCMP: Royal Canadian Mounted Police
T1: Pre-training
T2: Post-training (i.e., ~ 16 weeks after training commences)
T3: A follow-up 1 year after T2
UP: Unified Protocol for the Transdiagnostic Treatment of Emotional Disorders

## Footnotes

1 Pre-registered with aspredicted.org for the PSP PTSI Study and associated hypotheses occurred on March 7, 2022 with the name, “An Augmented Training Program for Preventing Post-Traumatic Stress Injuries Among Diverse Public Safety Personnel” (#90136).
