# Supplementary material for: Assessing the impact of the Royal Canadian Mounted Police (RCMP) protocol and Emotional Resilience Skills Training (ERST) among diverse public safety personnel
Source: BMC Psychol. 2022 Dec 9;10:295. doi: 10.1186/s40359-022-00989-0 (PMC9733219; doi:10.1186/s40359-022-00989-0)
Supplement: Supplementary file 1 — Additional files 1. Tables—PTSI_Protocol_Paper_Supplemental.pdf. [file 40359_2022_989_MOESM1_ESM.pdf]

Supplemental Tables and References for “Assessing the impact of the Royal Canadian Mounted Police (RCMP) Protocol and Emotional Resilience Skills Training (ERST) Among Diverse Public Safety Personnel”, under review for publication.

Supplemental Table 1. *PSP PTSI Study Recruitment and Data Collection Time Frame Overview*

Approximate Time Frames Relative to Sector Start[a]	Milestone	Assessment	Activity
Week 1	T1	N/A	On-Boarding Session – Group 1[b]
	T1	DA1	First Daily Assessment – Group 1 (i.e., "DA1")
	T1	FA1	First Full Survey – Group 1 (i.e., "F1")
Week 2	T1	FA1	First Clinical Interview – Group 1 (i.e., "C1")
	T1	N/A	On-Boarding Session – Group 2
	T1	DA1	First Daily Assessment – Group 2 (i.e., "DA1")
	T1	FA1	First Full Survey – Group 2 (i.e., "F1")
Week 3	T1	FA1	First Clinical Interview – Group 2 (i.e., "C1")
Week 4	T1	N/A	ERST Starts[c]
Week 5	T1	MA1	First Monthly Assessment (i.e., "MA1")
Weeks 7-17	T1	MA2-MA4	Monthly Assessments (i.e., "MA2-MA4")
Week 17	T1	N/A	ERST Training final week
Week 18	T2	FA2	Second Full Survey (i.e., "F2")
	T2	FA2	Second Clinical Interview (i.e., "C2")
Weeks 19-69	T2	MA5-MA17	Monthly Assessments (i.e., "MA5-MA17")
Weeks 70	T3	FA3	Third Full Survey (i.e., "F3")
	T3	FA3	Third Clinical Interview (i.e., "C3")

Notes. T1…T3 – Times 1 to 3; FA1…FA3 – Full Assessments 1 to 3; F1…F3 – Full Surveys 1 to 3; C1…C3 – Clinical Interviews 1 to 3; MA1…MA17 – Monthly Assessments 1 to 17; DA1…DA491 Daily Assessments 1 to ~491.

[a]Recruitment started in May 2021 and continued through March 2022. Participants from separate sectors (i.e., fire, police, paramedics, public safety communicators) were brought into the study in a staggered approach. Fire was onboarded in November 2021; police were onboarded in January 2022; paramedics were onboarded in February 2022; and public safety communicators were onboarded end of April 2022.

[b]Participants within each sector were on-boarded and interviewed in two separate groups to accommodate shift work and clinician workload.

[c]ERST Training sessions were offered twice weekly and occasionally more than 1 week apart due to shift work concerns. Trainers adhered to the above schedule as closely as possible, while accommodating shift work and the specific logistics needs of each sector community.

Supplemental Table 2. Posttraumatic Stress Injury Symptom Measures

	F1…F3	H1 Δ Across Milestone	MA1… MA17
Alcohol Use Disorders Identification Test (AUDIT)	•	F1>F2=F3	•
Cannabis Use Disorders Identification Test – Revised (CUDIT)	•	F1>F2=F3	•
Chronic Pain Questionnaire (CPQ)	•	F1>F2=F3	•
Depression Anxiety Stress Scale – 21 (DASS-21) – Stress Subscale	•	F1>F2=F3	•
Dyadic Adjustment Scale (DAS)	•	F1<F2=F3	
Expression of Moral Injury Scale – Military Version – Short Form (EMIS-M-SF)	•	F1>F2=F3	
Generalized Anxiety Disorder Scale – 7 (GAD-7)	•	F1>F2=F3	•
History of Anxiety, Mood, and Other Psychiatric Diagnoses (HAMOPD)	•	F1=F2=F3	
Insomnia Severity Index (ISI)	•	F1>F2=F3	•
Life Events Checklist – 5 (LEC-5)	•	F1<F2<F3	•
McGill Pain Questionnaire - Short Form (MPQ-SF)[a]	•	F1>F2=F3	•
Medications and Drug Use Scale (MDUS)	•	F1=F2=F3	•
Mental Health Continuum - Short Form (MHC-SF)	•	F1>F2=F3	•
Panic Disorder Severity Scale (PDSS)	•	F1>F2=F3	•
Patient Health Questionnaire - 9 (PHQ-9)	•	F1>F2=F3	•
PTSD Checklist for DSM-5 (PCL-5)	•	F1>F2=F3	•
Social Interaction Phobia Scale (SIPS)	•	F1>F2=F3	•
Tobacco Use Questionnaire (TUQ)	•	F1=F2=F3	•
Utrecht Work Engagement Scale - 9 (UWES-9)	•	F1<F2=F3	•

Notes: F1 – First Full Survey (i.e., T1 [i.e., milestone 1] - pre-training); F2 – Second Full Survey (i.e., T2 [i.e., milestone 2] - post-training); F3 Third Full Survey (i.e., T3 [i.e., milestones 3] – one-year follow up); MA1…MA17 – Monthly Assessments; H1 Δ – Hypothesized change over time; [a]Based on participant and clinician feedback, a short form of this scale was introduced after F1 for fire and for all milestones for police, paramedics, and public safety communicators.

Supplemental Table 3. Primary Differences Associated with Posttraumatic Stress Injuries

	F1…F3	H1 Δ Across Milestone	MA1… MA17
Anxiety Sensitivity Index-3 (ASI-3)	•	F1<F2=F3	
Beliefs about Emotions Scale (BES)	•	F1>F2=F3	
Brief Experiential Avoidance Questionnaire (BEAQ)	•	F1>F2=F3	
Brief Fear of Negative Evaluation - Straightforward Items (BFNE-S)	•	F1>F2=F3	
Brief Resilience Scale (BRS)	•	F1<F2=F3	•
Childhood Stressors Screen (CSS)[a]	F1 only		
Dimensions of Anger Reactions-5 (DAR-5)	•	F1>F2=F3	
Drinking Motives Questionnaire - Short Form (DMQ-SF)	•	F1>F2=F3	•
Emotion Regulation Questionnaire (ERQ)			
ERQ – Cognitive Reappraisal Subscale	•	F1<F2=F3	
ERQ – Emotion Suppression Subscale	•	F1>F2=F3	
HEXACO Personality Scales[a]			
Honesty-Humility	•	F1=F2=F3	
Emotionality	•	F1=F2=F3	
eXtraversion	•	F1=F2=F3	
Agreeableness (versus Anger)	•	F1<F2=F3	
Conscientiousness	•	F1=F2=F3	
Openness to Experience	•	F1<F2=F3	
Illness/Injury Sensitivity Index - Revised (ISI-R)	•	F1>F2=F3	
Intolerance of Uncertainty Scale - Short Form (IUS-12)	•	F1>F2=F3	
Pain Anxiety Symptom Scale-20 (PASS-20)	•	F1>F2=F3	
Southampton Mindfulness Questionnaire (SMQ)	•	F1<F2=F3	

Notes: F1 – First Full Survey (i.e., T1 [i.e., milestone 1] - pre-training); F2 – Second Full Survey (i.e., T2 [i.e., milestone 2] – post-training); F3 – Third Full Survey (i.e., T3 [i.e., milestones 3] - one-year follow up); MA1…MA17 – Monthly Assessments; H1 Δ – Hypothesized change over time; [a]Based on participant and clinician feedback, a short form of this scale was introduced after F1 for fire and for all milestones for police, paramedics, and public safety communicators.

Supplemental Table 4. Secondary Individual Differences Associated with Posttraumatic Stress Injuries

	F1…F3	H1 Δ Across Milestone	MA1… MA17
Canadian Armed Forces Recruit - Mental Health Service Use Questionnaire (CAFR-MHSUQ)	•	F1<F2=F3	•
Discrimination Questions	•	F1=F2=F3	
Institutional Support and Betrayal Questionnaire (ISBQ)			
ISBQ – Support Subscale[b]	•	F1<F2<F3	
ISBQ – Betrayal Subscale[a]	•	F1>F2>F3	
Mental Health Knowledge Schedule (MAKS)[b]	•	F1<F2=F3	
Opening Minds Survey for Workplace Attitudes (OMSWA)[a]	•	F1>F2=F3	
Parental Assessment of Childhood Stress (PACS)	•	F1>F2>F3	
Post Traumatic Growth Inventory – Short Form (PTGI-SF)	•	F1<F2<F3	
Public Safety Personnel Stressors (PSP-Stress)	•	F1>F2>F3	
Public Safety Officer Supports (PSP-Support)	•	F1=F2=F3	
RCMP Specific Support Scale (RSSS)	•	F1<F2=F3	
Self-Care and Mental Health Access for Public Safety (SCMHA-PS)	•	F1<F2=F3	•
Social Provision Scale - 10 (SPS-10)[b]	•	F1<F2=F3	
Unified Protocol Behavioural Avoidance Questionnaire (UPBAQ)[c,d]	•	F1>F2=F3	•
Unified Protocol Cognitive Questionnaire (UPCQ)[c]	•	F1<F2=F3	•
Unified Protocol Knowledge Acquisition (UPKA)	F2, F3 only	F2=F3	

Notes: F1 – First Full Survey (i.e., T1 [i.e., milestone 1] – pre-training); F2 – Second Full Survey (i.e., T2 [i.e., milestone 2] – post-training); F3 – Third Full Survey (i.e., T3 [i.e., milestones 3] – one-year follow up); MA1…MA17 – Monthly Assessments; H1 Δ – Hypothesized change over time; [a]Based on participant and clinician feedback, a short form of this scale was introduced after F1 for fire and for all milestones for police, paramedics, and public safety communicators; [b]Based on participant and clinician feedback, this scale was identified as redundant with other measures and removed after F1 for fire and from all police, paramedic, and public safety communicators milestone assessments; [c]A technical error resulted in this questionnaire being missed in the monthly assessments until April 15, 2022; [d]Due to a technical error, this questionnaire was missed at F1 for fire.

Supplemental Psychometrics and References for Self-Report Measures (Alphabetically)

Alcohol Use Disorders Identification Test (AUDIT; (1)). The AUDIT is a 10-item self-report questionnaire comprised of items assessing alcohol intake, alcohol dependence, and adverse consequences of alcohol use over the past 12 months. Items such as "How many drinks containing alcohol do you have on a typical day?" are reported on a 5-point Likert-type scale ranging from 0 (*never*) to 4 (*daily or almost daily*). A positive screen for AUD was determined based on total score (i.e., scores greater than 15 can be used to identify clinically significant hazardous drinking and dependence; (2)). Psychometric evaluation of the AUDIT has demonstrated good internal consistency (α = .85) and good test-retest reliability (*r* = .83 to .95) in the general population (3, 4) and in police populations (α = .81; (5)).

Anxiety Sensitivity Index-3 (ASI-3; (6)). The ASI-3 is an 18-item self-report measure assessing the tendency to fear anxiety symptoms based on the belief that they may have harmful consequences. Items such as "When my chest feels tight, I get scared that I won't be able to breathe properly," are rated on a 0 (*agree very little*) to 4 (*agree very much*) Likert scale. Higher scores indicate greater sensitivity to anxiety symptoms. Factor analysis supports a three-factor structure (i.e., somatic, cognitive, and social fears), which correspond to the three theorized dimensions of anxiety sensitivity (i.e., fear of somatic sensations, fear of cognitive dyscontrol, and fear of socially observable signs of anxiety, respectively). The ASI-3 has been found to have better factorial validity and internal consistency relative to the original Anxiety Sensitivity Index (7) and has displayed convergent, discriminant, and criterion validity (6). Psychometric evaluation of the ASI-3 has indicated good internal consistency (αs = .83, .86, and .79 for somatic, cognitive, and social fears subscales respectively, as well as α = .89 for the ASI-3 total score) and good test-retest reliability (*r*s = .45, .51, and .39 for somatic, cognitive, and social fears respectively, as well as *r* = .31 for the ASI-3 total score; (8)).

Beliefs about Emotions Scale (BES; (9)). The BES is a 12-item self-report scale designed to measure respondents' beliefs about the unacceptability of experiencing and expressing emotions. Each item is measured on a seven-point Likert scale, ranging from 0 (*totally disagree*) to 6 (*totally agree*). Higher scores indicate greater beliefs that it is unacceptable for respondents to experience or express emotion. The scale has high internal consistency (α = .91). Measures of dysfunctional attitudes, self-sacrifice, and problematic perfectionism, as well as symptoms of depression, anxiety, and fatigue, have also been significantly correlated with scores on the BES.

Brief Experiential Avoidance Questionnaire (BEAQ; (10)). The BEAQ is a 15-item self-report scale created to provide a shortened alternative to the Multidimensional Experiential Avoidance Questionnaire (MEAQ; (11)). The BEAQ assesses a broad range of experiential avoidance dimensions such as avoidance, psychopathology, and quality of life. Items such as "I'm quick to leave any situation that makes me feel uneasy," are rated on a 6-point Likert scale ranging from 1 (*strongly disagree*) to 6 (*strongly agree*). Higher scores indicate greater avoidance. Psychometrics evaluation of the BEAQ has demonstrated good internal consistency (α = .84) in veterans seeking outpatient treatment and adequate consistency (α = .77) in veterans seeking residential treatment for PTSD (12).

Brief Fear of Negative Evaluation Scale - Straightforward Items (BFNE-S; (13)). The BFNE-S is comprised of the eight straightforwardly worded items from the original BFNE (14) and assesses fears of negative evaluation with 5-point Likert scales from 0 (*not at all characteristic of me*) to 4 (*extremely characteristic of me*). Higher scores indicate greater fear of negative evaluation. Use of only the straightforward items has been ratified by recent comparative analyses (15). The BFNE-S has demonstrated excellent internal consistency, factorial validity, and construct validity in undergraduate (αs = .94 to .96; (16, 17)) and clinical (αs = .90 to .96; (13)) samples.

Brief Resilience Scale (BRS; (18)). The BRS is a 6-item self-report measure designed to assess resilience, or a person's ability to bounce back or recover from stress. Items such as "I tend to bounce back quickly after hard times," are rated on a scale from 1 (*strongly disagree*) to 5 (*strongly agree*). Higher scores indicate a greater sense of resiliency. The BRS has demonstrated good test-retest reliability and internal consistency (αs = .80 to .91) across clinical and non-clinical samples, and has been independently determined to be among the most psychometrically sound of available resilience measures (19).

Canadian Armed Forces Recruit - Mental Health Service Use Questionnaire (CAFR-MHSUQ; (20)). The CAF-R-MHSUQ is a 4-item self-report questionnaire designed to measure a participant's willingness to seek mental health services. Items such as "If I developed mental health problems, I would expect to seek mental health treatment from a professional," are rated on a 5-point Likert scale ranging from 1 (*strongly agree*) to 7 (*strongly disagree*). Higher scores indicate greater willingness to seek mental health services.

Cannabis Use Disorders Identification Test - Revised (CUDIT-R; (21, 22)). The CUDIT-R is an 8-item self-report questionnaire designed to measure cannabis use and misuse. Items such as "How often do you use cannabis?" are rated on a 5-point Likert scale ranging from 0 to 4, with anchors changing based on the item. A positive screen for CUD is determined based on total score (i.e., a cut-off score of 13 or higher indicating clinically significant hazardous use and dependence). The CUDIT-R is well supported, with high sensitivity (i.e., 91%) and specificity (i.e., 90%) for identifying problematic use (21, 22). Psychometric evaluation of the CUDIT-R has demonstrated good internal consistency among college students (α = .83; (23)).

Childhood Stressors Screen (CSS). The CSS is a 22-item self-report questionnaire designed to measure aversive childhood experiences. Items such as "When you were growing up, how often did your family run out of money or find it hard to pay for basic necessities life food or clothing?" are rated a Likert-style scale ranging from 0 (*never*) to 4 (*very often*). Most CSS items were derived from the Canadian Community Health Survey: Mental Health, 2012 (24). Items three, four, and five were derived from the Childhood Experiences of Violence Questionnaire (25). A shortened version was introduced at F1 for fire and for all police, paramedic, and public safety communicators milestone assessments based on a subset of items suggested by Afifi (personal communication, November 15, 2021). Items 1, 2, 5, and 14 to 22 were retained from the original version.

Chronic Pain Questionnaire (CPQ; (26)). The CPQ is a self-report questionnaire designed to measure the location, intensity, and duration of physical chronic pain. Items such as "Do you experience chronic pain?" are answered with face-valid options (e.g., yes, no). Additionally, items such as "What caused the chronic pain that most interfered with your life?" are rated on a Likert-like scale (e.g., Injury related to active duty, Injury related to work other than active duty). The CPQ is a new measure and psychometrics will be available as soon as possible.

Depression Anxiety Stress Scale - 21 (DASS; (27, 28)). The DASS is a 21-item self-report questionnaire designed to measure the negative emotional states of depression, anxiety, and stress. Given that symptoms of MDD and GAD were measured with other questionnaires (i.e., PHQ-9 and GAD-7), only the Stress subscale was used. The Stress subscale is sensitive to levels of chronic non-specific arousal and assesses difficulty relaxing, nervous arousal, and being easily upset/agitated, irritable/over-reactive and impatient. Respondents are asked to use 4-point severity/frequency scales ranging from 0 (*did not apply to me*) to 3 (*applied to me very much*) to rate the extent to which they have experienced each state over the past week. Higher scores indicate greater subjective experiences of stress. Psychometric assessment of the Stress subscale has indicated good internal consistency (α = .78; (29)) among medical students and excellent internal consistency (α = .91; (30)) among a community sample.

Dimensions of Anger Reactions - 5 (DAR-5; (31)). The DAR-5 is a 5-item questionnaire assessing participants' self-reported levels of anger. Items such as "When I got angry at someone, I wanted to hit them," are rated on a 1 (*none or almost none of the time*) to 5 (*all or almost all of the time*) Likert scale. Higher scores indicate greater self-reported levels of anger. The DAR-5 was adapted from the original Dimensions of Anger Reactions measure (32), which displayed strong psychometric properties but was lengthy and overcomplicated. The resulting DAR-5 has displayed concurrent validity with the commonly-used State Trait Anger Expression Inventory 2 (33) and predictive of changes in PTSD; further, it displays strong internal reliability (α = .90), a robust one-factor structure, and is recommended for screening anger in long questionnaire batteries (34).

Discrimination Questions. Institutional discrimination and harassment were evaluated using 4-items: 1) "Have you experienced sexual harassment in relation to your work (for example, in a work setting, from a colleague but outside of work, etc.)?"; 2) "Have you experienced sexual assault in relation to your work (for example, in a work setting, from a colleague but outside of work, etc.)?"; 3) "Have you experienced harassment (non-sexual) in relation to your work (for example, in a work setting, from a colleague but outside of work, etc.)?"; and 4) "Have you experienced discrimination in relation to your work (for example, in a work setting, from a colleague but outside of work, etc.)?". The items included face-valid response options (i.e., yes, no, prefer not to answer). Participants were also asked to identify the grounds on which they were discriminated against (e.g., race, sex, gender identity). The items were adapted from previous work done by the International Women's Media Foundation (https://www.iwmf.org/wp-content/uploads/2018/06/Violence-and-Harassment-against-Women-in-the-News-Media.pdf).

Drinking Motives Questionnaire - Short Form (DMQ-SF; (35)). The DMQ-SF is a 4-item self-report questionnaire that assesses motives for drinking behaviours. Items such as "How often do you use alcohol to manage physical pain?" are rated on a 5-point Likert scale ranging from 0 (*never*) to 4 (*daily or almost daily*). The DMQ was designed for large-scale screenings as part of demographic history and is entirely dependent on participant self-report.

Dyadic Adjustment Scale - 4 (DAS-4; (36)). The DAS-4 is a 4-item self-report questionnaire that assesses marital satisfaction. Items such as "In general, how often do you think that things between you and your partner are going well?" are rated on a 1 (*never*) to 6 (*all the time*) Likert scale. Higher scores indicate greater subjective marital satisfaction. The DAS-4 has displayed very good psychometric properties (36).

Emotion Regulation Questionnaire (ERQ; (37)). The ERQ is a 10-item questionnaire designed to assess how an individual regulates positive and negative emotions. The ERQ has two subscales: 1) Cognitive Reappraisal, which evaluates a participants ability to change their thinking (e.g., "When I want to feel more positive emotion (such as joy or amusement), I change what I'm thinking about."); and 2) Emotion Suppression, which evaluates the extent to which participants repress their emotions (e.g., "When I'm feeling negative emotions I make sure not to express them."). Items are rated on a 1 (*strongly disagree*) to 7 (*strongly agree*) Likert scale. The ERQ has displayed good internal reliability (αs range from .73 to .79) and test-retest reliability across three months (37), as well as a robust factor structure (37).

Expression of Moral Injury Scale – Military – Short Form (EMIS-M-SF; (38)). The EMIS-M-SF is a 4-item self-report measure designed to swiftly assess for warning signs of a moral injury in military populations. Items (e.g., "I feel guilt about things that happened during my military service that cannot be excused.") are rated on a 1 (*strongly disagree*) to 5 (*strongly agree*) Likert scale. Higher scores indicate greater experience of moral injury. The EMIS-M-SF has been psychometrically validated in a military sample, with good internal consistency (α = .84).

Generalized Anxiety Disorder Scale - 7 (GAD-7; (39)). The GAD-7 is a 7-item self-report measure assessing for symptoms of anxiety and worry. Participants are asked to rate their experiences of symptoms over the last two weeks (e.g., "Feeling nervous, anxious, or on edge") on a 0 (*not at all*) to 3 (*nearly every day*) Likert scale. A positive screen for generalized anxiety disorder (GAD) was determined based on total score (i.e., scores greater than 9 can be used to identify persons reporting clinically significant symptoms; (40)). The GAD-7 has good reliability, and construct, criterion, procedural, and factorial validity (39), as well as good internal consistency (α=.89) and inter-item correlations (.45-.65) in a community sample (41).

HEXACO Personality Inventory - 100-item scale (HEXACO-100; (42)). The HEXACO-100 is a self-report measure which corresponds to the six personality dimensions identified in the HEXACO model (43). Items such as "People sometimes tell me that I am too critical of others," are ranked on a 1 (*strongly disagree*) to 5 (*strongly agree*) Likert scale. The HEXACO model of personality is comprised of six personality dimensions: honesty/humility, emotionality, extraversion, agreeableness, conscientiousness, and openness (44). The HEXACO-100 is psychometrically sound, with good internal consistency in college and community samples (αs range from .81 to .85), inter-factor correlations ranging from |.02| to |.42|, and convergent validity with other measures of personality (42). Based on participant and clinician feedback, a shortened form of the HEXACO-100 (HEXACO-60; (45)) was introduced after F1 for fire and for all police, paramedic, and public safety communicators milestone assessments.

HEXACO Personality Inventory – 60-item scale (HEXACO-60; (45)). The HEXACO-60 is a short version of the 100-item HEXACO personality inventory, with 10 items from each of the 6 personality dimensions in the HEXACO model (43). Items (e.g., "I often push myself very hard when trying to achieve a goal.") are rated on a 1 (*strongly disagree*) to 5 (*strongly agree*) Likert scale. The HEXACO-60 has been psychometrically validated with acceptable internal consistency in both an undergraduate (αs range from .77 to .80) and a community sample (αs range from .73 to .80). In addition to the standard HEXACO-60 items, items 97 to 100 of the HEXACO-100 were retained, as these constitute an interstitial facet absent from the standard 60-item version. The HEXACO-60 plus these four interstitial facet items replaced the HEXACO-100 after F1 for fire and for all police, paramedic, and public safety communicators milestone assessments.

Illness/Injury Sensitivity Index - Revised (ISI-R). The ISI-R is 9-item revision of the original Illness/Injury Sensitivity Index (46) designed to measure fears of illness and injury (e.g., "I worry about my physical health."). Items are rated on a 5-point Likert scale ranging from 0 (*agree very little*) to 4 (*agree very much*). Two factors, Fear of Illness (e.g., "I worry about becoming physically ill.") and Fear of Injury (e.g., "I am frightened of being injured."), are represented within the ISI-R (47); however, the total summed score is used in most analyses, with higher scores indicating greater fear. The ISI-R has excellent internal consistency ($\alpha = .86$), convergent validity with other measures related to injury and illness ($r > .65$), and correlates highly with the original index, $r = .96$ (48).

Insomnia Severity Index (ISI; (49)). The ISI is a 7-item self-report questionnaire that assesses difficulties with falling or staying asleep. Items such as "How satisfied/dissatisfied are you with your current sleep pattern?" are rated on a 5-point Likert scale ranging from 0 (*very satisfied*) to 4 (*very dissatisfied*). Higher scores indicate greater sleep difficulties. The ISI has solid psychometric support including adequate internal consistency (i.e., $\alpha = .74$ to .78), sensitivity (94%), specificity (94%), and convergent validity (50).

Institutional Betrayal and Support Questionnaire (IBSQ; (51-53)). The IBSQ is a 29-item self-report questionnaire that assesses for feelings of support versus lack of support by an institution. The questionnaire was modified to measure perceptions of support received by the PSP following exposures to diverse potentially psychologically traumatic events (PPTEs). PSP are asked whether their organization

played a role following exposure by responding in any of several different ways, such as “Meeting your needs for support and accommodations,” and “Responding inadequately to the experience/s, if reported.” Response options include yes, no, and N/A. Preliminary research with an earlier version of this measure has revealed a one-factor solution. The questionnaire has been modified to ask specifically about experiences with the institution participants are employed by at the time of research (51, 52). Based on participant and clinician feedback, a short form of the ISBQ (IBQ2; (54)) was introduced after F1 for fire and for all police, paramedic, and public safety communicators milestone assessments.

Institutional Betrayal Questionnaire – 2 (IBQ2; (55)). The IBQ2 is a 12-item self-report questionnaire that measures feelings of betrayal towards an institution after experiencing a potentially psychologically traumatic event (e.g., sexual assault, motor vehicle accident, sudden death). Respondents are asked to consider larger institutions (e.g., church, military unit, workplace) to which they belong or have belonged and consider whether or not the institution played a role in a previously identified event. Items include various actions an organization could take such as “Not taking proactive steps to prevent this type of experience,” and “Denying your experience in some way.” Response options include yes, no, and N/A. The IBQ2 has been validated in a sample of sexual assault survivors and showed good convergent and discriminant validity (55). The IBQ2 replaced the ISBQ after F1 for fire and for all police, paramedic, and public safety communicators milestone assessments.

Intolerance of Uncertainty Scale - Short Form (IUS-12; (56)). The IUS-12 is a 12-item questionnaire that measures responses to uncertainty, ambiguous situations, and the future. Items are rated on a 5-point Likert scale ranging from 1 (*not at all characteristic of me*) to 5 (*entirely characteristic of me*). Higher scores indicate greater intolerance of uncertainty. The IUS-12 has a continuous latent structure and has two factors (56, 57), prospective IU (7 items; e.g., “I can’t stand being taken by surprise.”) and inhibitory IU (5 items; e.g., “When it’s time to act, uncertainty paralyses me.”). The IUS-12 has sound psychometric properties (56, 58) and strong internal consistency for the total and subscale scores (αs range from .85 to .91; (56)).

Life Event Checklist for DSM–5 (LEC-5; (59, 60)). The LEC-5 is a commonly used tool for assessing self-reported exposures to diverse PPTE. The LEC-5 presents respondents with 17 different PPTE types each with six response options including happened to me, witnessed it, learned about it, part of my job, not sure, or doesn’t apply (60). LEC for DSM–IV has demonstrated good convergent and discriminant validity, test–retest reliability over a 7-day period, and concurrent validity with other measures of PPTE exposures (59, 61). The only difference between LEC-5 and the LEC for DSM-IV is that the LEC-5 allows respondents to report PPTE exposures that occurred “as part of my job,” which corresponds with contemporary PTSD diagnostic criteria (62). In the current study, participants were asked specifically about PPTE exposures that occurred “as part of my public safety job,” to avoid confounds with other employment. For the First Full Survey participants were asked to report on PPTE during their “entire life (growing up, as well as adulthood)”; in contrast, for subsequent surveys, participants were asked to report on PPTE “since you last completed this questionnaire.”

McGill Pain Questionnaire - Short Form (MPQ-SF; (63)). The MPQ-SF is a commonly used tool for the measurement of pain experience. The MPQ-SF includes a pain rating index (PRI) of 15 of the most commonly used adjectives that describe sensory and affective aspects of pain (64). The MPQ-SF also includes a visual analogue scale (VAS) to help assess pain intensity. The checklist is rated on a 4-point intensity scale ranging from 0 (*none*) to 3 (*severe*). The MPQ-SF has been found to correlate highly with the original MPQ (64), and demonstrates good factorial validity for both sensory and affective components (.78 and .76 respectively; (64)). Based on participant and clinician feedback, the RPI and PPI were removed after F1 for fire and from all police, paramedic, and public safety communicators milestone assessments, retaining only the VAS.

Medications and Drug Use Scale (MDUS; Carleton, Duranceau, & LeBouthillier, 2016; unpublished scale). The MDUS is a 5-item self-report questionnaire that assesses self-reported use of medications and drugs not otherwise assessed by demographics. Items such as “Do you regularly use any prescription or over-the-counter medications?” are responded to with face-valid options (e.g., yes, no). The MDUS was designed for large-scale screenings as part of demographic history and is entirely dependent on participant self-report.

Mental Health Continuum - Short Form (MHC-SF; (65-67)). The MHC-SF is a 14-item scale designed to measure emotional, psychological, and social well-being. The MHC-SF was derived as a shortened version of the Mental Health Continuum Long Form and has good internal reliability (α = .74; (65)). The MCH-SF measures the degree of 1) emotional well-being, 2) psychological well-being, and 3) social well-being (65). Items are rated on a 6-point Likert scale ranging from 0 (*never*) to 5 (*every day*). Higher scores indicate greater perceived well-being in each of the three areas. The MHC-SF has also demonstrated good internal reliability in French (αs for subscales range from .78 to .90; (67)) and in Dutch (α = .89 and αs for subscales range from .74 to .83; (66)).

Mental Health Knowledge Schedule (MAKS; (68)). The MAKS is a 15-item self-report questionnaire designed to measure mental health literacy and stigma. Items such as “Most people with mental health problems want to have paid employment,” are rated on a 5-point Likert scale ranging from 1 (*strongly disagree*) to 5 (*strongly agree*). Higher scores indicate greater mental health literacy. The MAKS is a relatively new measure, but the available psychometric data support the internal consistency (α = .65) and test-retest reliability (.57 to .87) of the measure (68); in addition, the measure appears sensitive to changes based on interventions (69). Based on participant and clinician feedback, the MAKS was identified as redundant with other measures and was removed after F1 for fire and from all police, paramedic, and public safety communicators milestone assessments.

Opening Minds Survey of Workplace Attitudes (OMSWA; (70)). The OMSWA is a 22-item self-report questionnaire designed to measure mental health stigma and workplace attitudes. Items such as “I would be upset if a co-worker with a mental illness always sat next to me at work,” are rated on a 5-point Likert scale ranging from 1 (*strongly disagree*) to 5 (*strongly agree*). Higher scores indicate greater stigmatizing attitudes towards mental health conditions in the workplace. The OMSWA is a relatively new measure, but the available psychometric data support the internal consistency of the measure (70). The measure is currently in use as a standard metric by the Mental Health Commission of Canada. Based on participant and clinician feedback, a short form of the OMSWA was introduced after F1 for fire and for all police, paramedic, and public safety communicators milestone assessments.

Opening Minds Survey of Workplace Attitudes – Short Form (OMSWA-SF; (71)). The OMSWA-SF is a 9-item self-report questionnaire designed to briefly assess stigmatizing attitudes in the workplace. Items (e.g., “I would not be close friends with a co-worker who I knew had mental illness.”) are rated on a 1 (*strongly disagree*) to 5 (*strongly agree*) Likert scale. Higher scores indicate greater stigmatizing attitudes towards mental health conditions in the workplace. The OMSWA-SF has been psychometrically validated in a sample of PSP, with good internal consistency (α = .89). The OMSWA-SF replaced the OMSWA after F1 for fire and for all police, paramedic, and public safety communicators milestone assessments.

Pain Anxiety Symptoms Scale - 20 (PASS-20; (72)). The PASS-20 is a short form of the original Pain Anxiety Symptoms Scale (PASS; (73)) used to measure pain-related anxiety. Each of the 20 items (e.g., “When I feel pain I am afraid that something terrible will happen.”) are rated on a 6-point Likert scale ranging from 0 (*never*) to 5 (*always*). Each of four, 5-item subscales (i.e., Cognitive, e.g., “I can't think straight when in pain;” Fear, e.g., “Pain sensations are terrifying;” Escape/Avoidance, e.g., “I will stop any activity as soon as I sense pain coming on;” and Physiological, e.g., “Pain makes me nauseous.”)

provides a score that can be considered separately or, when summed, as a general measure of pain-related anxiety. Higher scores indicate greater pain-related anxiety. Factorial validity for both the total and subscale scores has been demonstrated for clinical (α = .83; (74)) and non-clinical samples (α = .91; (75)).

Panic Disorder Severity Scale (PDSS; (76)). The PDSS is a 7-item self-report measure designed to assess symptoms of panic disorder (e.g., "During the past week, were there any activities that you avoided or felt afraid of because they caused physical sensations like those you feel during panic attacks?"). The items assess panic frequency, distress during panic, panic-focused anticipatory anxiety, phobic avoidance of situations, phobic avoidance of physical sensations, impairment in work functioning, and impairment in social functioning. Items are rated on a 5-point Likert scale ranging from 0 (*none*) to 4 (*extreme*). A positive screen for panic disorder was determined based on total score (i.e., scores greater than 7 can be used to identify persons reporting clinically significant anxiety and distress; (77)). The self-report version of the PDSS has displayed excellent psychometrics, with one study finding strong internal validity (α = .92) and a correlation of .81 with the original measure (78).

Parental Assessment of Childhood Stress (PACS; Carleton, Duranceau, & Wright, 2016; unpublished scale). The PACS is a 26-item self-report questionnaire that assesses the degree to which a parent serving in public safety believes their child is experiencing distress that may be associated with the realities of such service. The PACS is a new measure and psychometrics will be available as soon as possible.

Patient Health Questionnaire - 9 (PHQ-9; (79)). The PHQ-9 is a 9-item self-report questionnaire that assesses symptoms of major depressive disorder (MDD). Items such as "Feeling down, depressed, or hopeless," are rated on a 4-point Likert scale ranging from 0 (*not at all*) to 3 (*nearly every day*). A positive screen for MDD was determined based on total score (i.e., scores greater than 9 can be used to identify persons reporting clinically significant distress; (80)). The PHQ-9 has solid psychometric support including sensitivity (88%), specificity (88%), and convergent validity (81, 82). Psychometric evaluation found the PHQ-9 to be a valid measure of depression symptoms and severity, with good internal consistency (α = .89) and test-retest reliability (r = .84) in the general population (79) and police populations (α = .88; (83)).

Posttraumatic Growth Inventory – Short Form (PTGI-SF; (84)). The PTGI-SF is a 10-item self-report questionnaire briefly assessing growth in response to traumatic events. Items (e.g., "I learned a great deal about how wonderful people are.") are rated on a 0 (*I did not experience this change as a result of my crisis*) to 5 (*I experienced this change to a very great degree as a result of my crisis*) Likert scale. Higher scores indicate greater posttraumatic growth. The PTGI-SF has been psychometrically validated in a large sample, with good internal consistency (α = .89) and can be reliably used in place of the full version with little loss of information.

PTSD Checklist for DSM-5 (PCL-5; (60)). The PCL-5 is a 20-item self-report measure used to assess symptoms of posttraumatic stress disorder (PTSD) experienced in the past month and to screen for persons reporting clinically-significant symptoms. Participants use a Likert scale ranging from 0 (*not at all*) to 4 (*extremely*) to rate how bothered they had been by different PTSD symptoms (e.g., "Repeated, disturbing memories, thoughts, or images of the stressful experience") over the past month. A positive screen for PTSD is determined based on total score (i.e., a score greater than 32 used to identify clinically significant symptoms), as well as meeting criteria on each individual symptom cluster (60). Psychometric evaluation has found the PCL-5 to be a reliable and valid measure of PTSD symptoms as described in the Diagnostic and Statistical Manual of Mental Disorders, 5th ed. (62), with strong internal consistency (α = .94) and test-retest reliability (r = .82) in PPTE-exposed populations (85).

Public Safety Personnel Stressors (PSP-Stress; (86)). The PSP-Stress is a 40-item self-report questionnaire designed to measure environmental stressors specific to public safety officers. The scale

was created by combining the 20-item Operational Police Stress Questionnaire (PSQ-Op) and the 20-item Organizational Police Stress Questionnaire (PSQ-Org). Items such as "The feeling that different rules apply to different people (e.g., favouritism)," are rated on a 7-point Likert scale ranging from 1 (*no stress at all*) to 7 (*a lot of stress*). Higher scores indicate greater subjective levels of stress. The PSP-Stress is a new measure and psychometrics will be available as soon as possible. The PSQ-Op scale has adequate reliability with a coefficient alpha of .93 and corrected item-total correlations ranging from .50 to .70. The PSQ-Org scale has adequate reliability with a coefficient alpha of .92 and corrected item-total correlations ranging from .41 to .73 (86).

Public Safety Personnel Support (PSP-Support; Carleton, 2015; unpublished scale). The PSP-Support is a 15-item self-report questionnaire designed to measure environmental supports specific to public safety officers. Items such as "Your family" are rated on a 5-point Likert scale ranging from 1 (*I feel undermined*) to 5 (*I feel as supported as I could ever hope to be*). Higher scores indicate greater subjective levels of support. The PSP-Support is a new measure and psychometrics will be available as soon as possible.

RCMP Specific Support Scale (RSSS; Krätzig, Jones, Hozempa, & Carleton, 2018; unpublished scale). The RSSS is a 14-item self-report questionnaire designed to measure environmental supports specific to Royal Canadian Mounted Police members. Items were adapted to fit all PSP for the current study (e.g., changing RCMP officer to PSP). Items such as "Do you feel that your supervisor would support you if you developed a mental illness/injury?" are rated on a 5-point Likert-type scale ranging from 1 (*strongly disagree*) to 5 (*strongly agree*). The RSSS is a new measure and psychometrics will be available as soon as possible.

Self-Care and Mental Health Access for Public Safety (SCMHA-PS; Carleton, Duranceau, & LeBouthillier, 2016; unpublished scale). The SCMHA-PS is a 25-item self-report questionnaire designed to measure environmental supports specific to public safety officers. The questionnaire contains two scales and one open-ended question. Items such as "Spouse" are rated on a 7-point Likert scale ranging from 1 (*I can and would access as an early resource*) to 7 (*I don't know if I have access*). The questionnaire contains one item with multiple open-ended sub-questions which asks, "How many days per week do you do each of the following activities?" There are 10 sub-questions with items such as "Socializing with other First Responders or other Public Safety Personnel?" The questionnaire also contains items such as "Contact mental health professionals for well-being (e.g., psychologists, therapists)" which are rated on a 5-point Likert scale ranging from 1 (*never*) to 5 (*annually*). The SCMHA-PS is a new measure and psychometrics will be available as soon as possible.

Social Interaction Phobia Scale (SIPS; (87)). The SIPS is a 14-item self-report measure designed to assess symptoms specific to social anxiety disorder (SAD; e.g., "When mixing socially I am uncomfortable."). Each item is measured on a 5-point Likert scale, ranging from 0 (*not at all characteristic of me*) to 4 (*entirely characteristic of me*). Higher scores indicate greater symptoms of social anxiety. The items were derived as a subset of items from the Social Interaction Anxiety and Social Phobia Scales (88). The SIPS is designed to measure three symptom dimensions of SAD: social interaction anxiety; fear of overt evaluation; and fear of attracting attention. SIPS total and subscale scores account for equivalent or greater variance relative to the original SIAS and SPS total scores (87). The SIPS total score has demonstrated excellent internal consistency ($\alpha = .92$) with adequate internal consistency (αs = .76 to .86) exhibited by all three sub-scales among undergraduate students (89). Similar results were found among patients with principal SAD and principal GAD patients, and slightly lower values but still good internal consistency were observed among healthy control sample (90). Use of the total score typically provides sufficient sensitivity and specificity for discerning clinical and nonclinical samples (i.e., scores greater than 20 can be used to identify persons reporting clinically significant distress). Subsequent research has replicated the psychometric properties, as well as convergent and

discriminant validity, of the SIPS in a large and independent sample (89). The SIPS is included as a measure of dimensional SAD symptoms (87, 91).

Social Provisions Scale - 10 (SPS-10; (92, 93). The SPS is a 10-item short form of the original measure designed to measure perceived social support (92). Items such as "There are people I can depend on if I really need it," are rated on a 4-point Likert scale ranging from 1 (*strongly disagree*) to 4 (*strongly agree*). Higher scores indicate greater feelings of social support. The SPS-10 has demonstrated excellent internal consistency (α = .88), concurrent validity (r = .93), and factorial validity (92). Based on participant and clinician feedback, the SIPS was identified as redundant with other measures and removed after F1 for fire and from all police, paramedic, and public safety communicators milestone assessments.

Southampton Mindfulness Questionnaire (SMQ; (94)). The SMQ is designed to provide a measure of mindful awareness of distressing thoughts and images. The SMQ is a 16-item scale with items such as, "Usually when I experience distressing thoughts and images, I am able to accept the experience," rated on a 7-point Likert scale ranging from 0 (*strongly disagree*) to 6 (*strongly agree*). Higher scores indicate greater ability to manage emotional reactions to distressing thoughts and images. Psychometric properties of the SMQ have exhibited an excellent internal consistency (α = .89) for the total sample and an acceptable consistency for the community (α = .89) and clinical (α = .82) groups; with corrected item-total correlations of r = .54 (94).

Tobacco Use Questionnaire (TUQ; (95)). The TUQ is an 8-item self- report questionnaire that assesses self-reported tobacco use. Items such as "Do you use tobacco (e.g., cigarettes, smokeless)?" are responded to with face-valid options (e.g., yes, no). The TUQ was designed for large-scale screenings as part of demographic history and is entirely dependent on participant self-report. Items were derived following review of the World Health Organization's Global Adult Tobacco Survey (95).

Unified Protocol Behavioral Avoidance Questionnaire (UPBAQ; (96)): The UPBAQ is a 5-item measure that was developed explicitly to assess the skill of approach-oriented behavior as it is taught in the Unified Protocol (UP). Participants rated items (e.g., "The way I acted in situations was driven by my distressing emotions," "I tried to avoid distressing emotions by avoiding situations that might cause them.)" by indicating how often they use each skill on a scale from 1 (*never*) to 5 (*always or when needed*). Higher scores indicate greater levels of emotional avoidance. The UPBAQ has demonstrated good internal consistency and validity (97).

Unified Protocol Cognitive Questionnaire (UPCQ; (98)). The UPCQ is a 7-item measure that was developed explicitly to assess the skill of cognitive flexibility as it is taught in the Unified Protocol (UP) as existing questionnaires assessing cognitive coping either included skills that are not emphasized in the UP or excluded key concepts covered in this module. Participants rated items (e.g., "I evaluated my thinking when I experienced a distressing emotion," "I understood that my thoughts can have an effect on my feelings and behaviors.") by indicating how often they use each skill on a scale from 1 (*never*) to 5 (*always or when needed*). Higher scores indicate greater use of cognitive coping skills. The UPCQ has demonstrated good internal consistency and validity (97).

Unified Protocol Knowledge Acquisition (UPKA; (97)). The UPKA questionnaire consists of 13 true/false items designed to assess core concepts taught during the PSP Emotional Resilience Skills Program. Example items include, "The goal of your emotional resilience course is to learn how to eliminate unwanted emotions like fear, anxiety, and sadness," and "How we currently feel can affect the way we interpret many situations." Several randomized controlled trials have assessed the efficacy of the psychological intervention on which the PSP ERST was based (e.g., (98)).

Utrecht Work Engagement Scale - 9 (UWES-9; (99)). The UWES-9 is a 9-item questionnaire assessing a person's work-related state of fulfillment. Questions such as "I am proud of the work that I do," are rated on a 6-point scale from 0 (*never*) to 6 (*always*). Higher scores indicate greater levels of work-related fulfillment. The UWES-9 has evidenced adequate internal consistency (αs = .75 to .85) and a more temporally stable factor structure than longer versions of the UWES (100).

References

1. Saunders JB, Aasland OG, Babor TF, Delafuente JR, Grant M. Development of the Alcohol-Use Disorders Identification Test (Audit) - Who Collaborative Project on Early Detection of Persons with Harmful Alcohol-Consumption. Addiction. 1993;88(6):791-804.
2. Gache P, Michaud P, Landry U, Accietto C, Arfaoui S, Wenger O, et al. The Alcohol Use Disorders Identification Test (AUDIT) as a screening tool for excessive drinking in primary care: Reliability and validity of a French version. Alcoholism-Clinical and Experimental Research. 2005;29(11):2001-7.
3. Daeppen JB, Yersin B, Landry U, Pecoud A, Decrey H. Reliability and validity of the Alcohol Use Disorders Identification Test (AUDIT) imbedded within a general health risk screening questionnaire: results of a survey in 332 primary care patients. Alcohol Clin Exp Res. 2000;24(5):659-65.
4. Reinert DF, Allen JP. The alcohol use disorders identification test: An update of research findings. Alcohol Clin Exp Res. 2007;31(2):185-99.
5. Davey JD, Obst PL, Sheehan MC. Developing a profile of alcohol consumption patterns of police officers in a large scale sample of an Australian police service. Eur Addict Res. 2000;6(4):205-12.
6. Taylor S, Zvolensky MJ, Cox BJ, Deacon B, Heimberg RG, Ledley DR, et al. Robust dimensions of anxiety sensitivity: Development and initial validation of the Anxiety Sensitivity Index-3. Psychol Assess. 2007;19(2):176-88.
7. Peterson RA, Reiss S. Anxiety Sensitivity Index Manual. 2nd ed. Worthington, OH: International Diagnostic Systems; 1992.
8. Osman A, Gutierrez PM, Smith K, Fang Q, Lozano G, Devine A. The Anxiety Sensitivity Index-3: analyses of dimensions, reliability estimates, and correlates in nonclinical samples. J Pers Assess. 2010;92(1):45-52.
9. Rimes KA, Chalder T. The Beliefs about Emotions Scale: validity, reliability and sensitivity to change. Journal of psychosomatic research. 2010;68(3):285-92.
10. Gamez W, Chmielewski M, Kotov R, Ruggero C, Suzuki N, Watson D. The brief experiential avoidance questionnaire: development and initial validation. Psychol Assess. 2014;26(1):35-45.
11. Gamez W, Chmielewski M, Kotov R, Ruggero C, Watson D. Development of a measure of experiential avoidance: the Multidimensional Experiential Avoidance Questionnaire. Psychol Assess. 2011;23(3):692-713.
12. Byllesby BM, Stayton Coe LE, Dickstein BD, Chard KM. Psychometric evaluation of the Brief Experiential Avoidance Questionnaire among treatment-seeking veterans with posttraumatic stress disorder. Psychological Trauma: Theory, Research, Practice, and Policy. 2020;12(7):785-9.
13. Weeks JW, Heimberg RG, Fresco DM, Hart TA, Turk CL, Schneier FR, et al. Empirical Validation and Psychometric Evaluation of the Brief Fear of Negative Evaluation Scale in Patients With Social Anxiety Disorder. Psychol Assess. 2005;17(2):179-90.
14. Leary MR. A brief version of the Fear of Negative Evaluation Scale. Personality and Social Psychology Bulletin. 1983;9:371-5.

15. Carleton RN, Collimore KC, McCabe RE, Antony MM. Addressing revisions to the Brief Fear of Negative Evaluation scale: Measuring fear of negative evaluation across anxiety and mood disorders. J Anxiety Disord. 2011;25(6):822-8.
16. Rodebaugh TL, Holaway RM, Heimberg RG. The treatment of social anxiety disorder. Clin Psychol Rev. 2004;24(7):883-908.
17. Carleton RN, Collimore KC, Asmundson GJG. Social anxiety and fear of negative evaluation: construct validity of the BFNE-II. J Anxiety Disord. 2007;21(1):131-41.
18. Smith BW, Dalen J, Wiggins K, Tooley E, Christopher P, Bernard J. The brief resilience scale: Assessing the ability to bounce back. International journal of behavioral medicine. 2008;15(3):194-200.
19. Windle G, Bennett KM, Noyes J. A methodological review of resilience measurement scales. Health Qual Life Outcomes. 2011;9:8.
20. Zamorski MA, Bennett RE, Boulos D, Garber BG, Jetly R, Sareen J. The 2013 Canadian Forces Mental Health Survey: Background and Methods. Canadian journal of psychiatry. 2016;61(1 Suppl):10S-25S.
21. Adamson SJ, Kay-Lambkin FJ, Baker AL, Lewin TJ, Thornton L, Kelly BJ, et al. An improved brief measure of cannabis misuse: The Cannabis Use Disorders Identification Test-Revised (CUDIT-R). Drug Alcohol Depend. 2010;110(1-2):137-43.
22. Guillem E, Notides C, Debray M, Vorspan F, Musa C, Leroux M, et al. Psychometric Properties of the Cannabis Use Disorders Identification Test in French Cannabis Misusers. J Addict Nurs. 2011;22(4):214-23.
23. Schultz NR, Bassett DT, Messina BG, Correia CJ. Evaluation of the psychometric properties of the cannabis use disorders identification test - revised among college students. Addict Behav. 2019;95:11-5.
24. Statistics Canada. Canadian Community Health Survey. Statistics Canada. 2012.
25. Walsh CA, MacMillan HL, Trocme N, Jamieson E, Boyle MH. Measurement of victimization in adolescence: development and validation of the Childhood Experiences of Violence Questionnaire. Child Abuse Negl. 2008;32(11):1037-57.
26. Carleton RN, Afifi TO, Turner S, Taillieu T, El-Gabalawy R, Sareen J, et al. Chronic Pain Among Public Safety Personnel in Canada. Canadian Journal of Pain. 2017;1(1):237-46.
27. Lovibond PF, Lovibond SH. The structure of negative emotional states: comparison of the Depression Anxiety Stress Scales (DASS) with the Beck Depression and Anxiety Inventories. Behav Res Ther. 1995;33(3):335-43.
28. Zlomke KR. Psychometric properties of internet administered versions of Penn State Worry Questionnaire (PSWQ) and Depression, Anxiety, and Stress Scale (DASS). Computers in Human Behavior. 2009;25:841-3.
29. Coker AO, Coker OO, Sanni D. Psychometric properties of the 21-item Depression Anxiety Stress Scale (DASS-21). African Research Review. 2018;12(2):135-42.
30. Antony MM, Bieling PJ, Cox BJ, Enns MW, Swinson RP. Psychometric properties of the 42-item and 21-item versions of the Depression Anxiety Stress Scales in clinical groups and a community sample. Psychol Assess. 1998;10:176-81.
31. Hawthorne G, Mouthaan J, Forbes D, Novaco RW. Response categories and anger measurement: do fewer categories result in poorer measurement?: development of the DAR5. Soc Psychiatry Psychiatr Epidemiol. 2006;41(2):164-72.

32. Novaco RW. Anger control: The development and evaluation of an experimental treatment. Oxford, England: Lexington; 1975. xii, 134-xii, p.
33. Tibubos AN, Schermelleh-Engel K, Rohrmann S. Short form of the State-Trait Anger Expression Inventory-2. European Journal of Health Psychology. 2020;27(2):55-65.
34. Forbes D, Alkemade N, Mitchell D, Elhai JD, McHugh T, Bates G, et al. Utility of the Dimensions of Anger Reactions-5 (DAR-5) scale as a brief anger measure. Depress Anxiety. 2014;31(2):166-73.
35. Kuntsche E, Kuntsche S. Development and validation of the Drinking Motive Questionnaire Revised Short Form (DMQ-R SF). J Clin Child Adolesc Psychol. 2009;38(6):899-908.
36. Sabourin S, Valois P, Lussier Y. Development and validation of a brief version of the Dyadic Adjustment Scale with a nonparametric item analysis model. Psychol Assess. 2005;17(1):15-27.
37. Gross JJ, John OP. Individual differences in two emotion regulation processes: implications for affect, relationships, and well-being. J Pers Soc Psychol. 2003;85(2):348-62.
38. Currier JM, Isaak SL, McDermott RC. Validation of the Expressions of Moral Injury Scale-Military version-Short Form. Clinical psychology & psychotherapy. 2020;27(1):61-8.
39. Spitzer RL, Kroenke K, Williams JB, Lowe B. A brief measure for assessing generalized anxiety disorder: the GAD-7. Arch Intern Med. 2006;166(10):1092-7.
40. Swinson RP. The GAD-7 scale was accurate for diagnosing generalised anxiety disorder. Evid Based Med. 2006;11(6):184.
41. Lowe B, Decker O, Muller S, Brahler E, Schellberg D, Herzog W, et al. Validation and standardization of the Generalized Anxiety Disorder Screener (GAD-7) in the general population. Med Care. 2008;46(3):266-74.
42. Lee K, Ashton MC. Psychometric Properties of the HEXACO-100. Assessment. 2018;25(5):543-56.
43. Lee K, Ashton MC. Psychometric properties of the HEXACO Personality Inventory. Multi Behav Res. 2004;39:329-58.
44. Ashton MC, Lee K, Goldberg LR, de Vries RE. Higher order factors of personality: do they exist? Personality and social psychology review : an official journal of the Society for Personality and Social Psychology, Inc. 2009;13(2):79-91.
45. Ashton MC, Lee K. The HEXACO-60: a short measure of the major dimensions of personality. J Pers Assess. 2009;91(4):340-5.
46. Taylor S. The structure of fundamental fears. J Behav Ther Exp Psychiatry. 1993;24(4):289-99.
47. Carleton RN, Asmundson GJG, Taylor S. Fear of Physical Harm: Factor Structure and Psychometric Properties of the Injury/Illness Sensitivity Index. Journal of Psychopathology and Behavioral Assessment. 2005;27(4):235-41.
48. Carleton RN, Park I, Asmundson GJG. The Illness/Injury Sensitivity Index: an examination of construct validity. Depress Anxiety. 2006;23(6):340-6.
49. Morin CM. Insomnia: Psychological assessment and management. New York, NY: Guilford Press; 1993.

50. Smith MT, Wegener ST. Measures of sleep: The Insomnia Severity Index, Medical Outcomes Study (MOS) Sleep Scale, Pittsburgh Sleep Diary (PSD), and Pittsburgh Sleep Quality Index (PSQI). Arthritis Care Res (Hoboken). 2003;49:S184-S96.
51. Smith CP, Freyd JJ. Dangerous safe havens: institutional betrayal exacerbates sexual trauma. J Trauma Stress. 2013;26(1):119-24.
52. Smith CP, Freyd JJ. Institutional Betrayal Questionnaire (IBQ) and Institutional Support and Betrayal Questionnaire (ISBQ) 2015 [Available from: http://dynamic.uoregon.edu/jjf/institutionalbetrayal/ibq.html.
53. Rosenthal MN, Smidt AM, Freyd JJ. Still second class: Sexual harassment of graduate students. Psychol Women Quart. 2016;40:364-77.
54. Smith CP, Freyd JJ. Insult, then injury: Interpersonal and institutional betrayal linked to health and dissociation. Journal of Aggression, Maltreatment, & Trauma. 2017;26:1117-31.
55. Reffi AN, Pinciotti CM, Orcutt HK. Psychometric Properties of the Institutional Betrayal Questionnaire, Version 2: Evidence for a Two-Factor Model. J Interpers Violence. 2021;36(11-12):5659-84.
56. Carleton RN, Norton PJ, Asmundson GJG. Fearing the unknown: a short version of the Intolerance of Uncertainty Scale. J Anxiety Disord. 2007;21(1):105-17.
57. McEvoy PM, Mahoney AE. Achieving certainty about the structure of intolerance of uncertainty in a treatment-seeking sample with anxiety and depression. J Anxiety Disord. 2011;25:112-22.
58. Khawaja NG, Yu L, N. H. A comparison of the 27-item and 12-item intolerance of uncertainty scales. Clin Psychol-Uk. 2010;14(3):97-106.
59. Gray MJ, Litz BT, Hsu JL, Lombardo TW. Psychometric properties of the life events checklist. Assessment. 2004;11:330-41.
60. Weathers FW, Litz BT, Keane TM, Palmieri PA, Marx BP, Schnurr PP. The PTSD Checklist for DSM-5 (PCL-5). Scale available from the National Center for PTSD: National Center for PTSD; 2013 [Available from: www.ptsd.va.gov.
61. Bae H, Kim D, Koh H, Kim Y, Park JS. Psychometric properties of the life events checklist-korean version. Psychiat Invest. 2008;5:163-7.
62. American Psychiatric Association. Diagnostic and Statistical Manual of Mental Disorders. 5th ed. Washington, DC: Author; 2013.
63. Melzack R. The short-form McGill Pain Questionnaire. Pain. 1987;30(2):191-7.
64. Wright KD, Asmundson GJG, McCreary DR. Factorial validity of the short-form McGill pain questionnaire (SF-MPQ). European Journal of Pain. 2001;5(3):279-84.
65. Keyes CL, Wissing M, Potgieter JP, Temane M, Kruger A, van Rooy S. Evaluation of the mental health continuum-short form (MHC-SF) in setswana-speaking South Africans. Clinical psychology & psychotherapy. 2008;15(3):181-92.
66. Lamers SM, Westerhof GJ, Bohlmeijer ET, ten Klooster PM, Keyes CL. Evaluating the psychometric properties of the Mental Health Continuum-Short Form (MHC-SF). Journal of clinical psychology. 2011;67(1):99-110.
67. Dore I, O'Loughlin JL, Sabiston CM, Fournier L. Psychometric Evaluation of the Mental Health Continuum-Short Form in French Canadian Young Adults. Canadian journal of psychiatry. 2017;62(4):286-94.

68. Evans-Lacko S, Little K, Meltzer H, Rose D, Rhydderch D, Henderson C, et al. Development and Psychometric Properties of the Mental Health Knowledge Schedule. Can J Psychiat. 2010;55(7):440-8.
69. Hansson L, Markstrom U. The effectiveness of an anti-stigma intervention in a basic police officer training programme: a controlled study. BMC Psychiatry. 2014;14.
70. Szeto ACH, Luong D, Dobson KS. Does labelling matter?: An examination of attitudes and perceptions of labels for mental disorders. Soc Psychiatry Psychiatr Epidemiol. 2013;48:659-71.
71. Boehme BAE, Shields RE, Asmundson GJG, Szeto AHC, Dobson KS, Carleton RN. A short version of the Opening Minds Scale for Workplace Attitudes: Psychometric properties in a sample of Canadian public safety personnel. Manuscript in Preparation.
72. McCracken LM, Dhingra L. A short version of the Pain Anxiety Symptoms Scale (PASS-20): preliminary development and validity. Pain Research and Management. 2002;7(1):45-50.
73. McCracken LM, Zayfert C, Gross RT. The Pain Anxiety Symptoms Scale: development and validation of a scale to measure fear of pain. Pain. 1992;50(1):67-73.
74. Coons MJ, Hadjistavropoulos HD, Asmundson GJG. Factor structure and psychometric properties of the Pain Anxiety Symptoms Scale-20 in a community physiotherapy clinic sample. European Journal of Pain. 2004;8(6):511-6.
75. Abrams MP, Carleton RN, Asmundson GJG. An exploration of the psychometric properties of the PASS-20 with a nonclinical sample. The Journal of Pain. 2007;8(11):879-86.
76. Shear MK, Brown TA, Barlow DH, Money R, Sholomskas DE, Woods SW, et al. Multicenter collaborative Panic Disorder Severity Scale. American Journal of Psychiatry. 1997;154(11):1571-5.
77. Shear MK, Rucci P, Williams J, Frank E, Grochocinski V, Vander Bilt J, et al. Reliability and validity of the Panic Disorder Severity Scale: Replication and extension. J Psychiatr Res. 2001;35(5):293-6.
78. Houck PR, Spiegel DA, Shear MK, Rucci P. Reliability of the self-report version of the panic disorder severity scale. Depress Anxiety. 2002;15(4):183-5.
79. Kroenke K, Spitzer RL, Williams JB. The PHQ-9: validity of a brief depression severity measure. J Gen Intern Med. 2001;16(9):606-13.
80. Manea L, Gilbody S, McMillan D. A diagnostic meta-analysis of the Patient Health Questionnaire-9 (PHQ-9) algorithm scoring method as a screen for depression. Gen Hosp Psychiatry. 2015;37:67-75.
81. Lowe B, Grafe K, Zipfel S, Witte S, Loerch B, Herzog W. Diagnosing ICD-10 depressive episodes: Superior criterion validity of the patient health questionnaire. Psychother Psychosom. 2004;73(6):386-90.
82. Lowe B, Unutzer J, Callahan CM, Perkins AJ, Kroenke K. Monitoring depression treatment outcomes with the patient health questionnaire-9. Medical Care. 2004;42(12):1194-201.
83. Pietrzak RH, Schechter CB, Bromet EJ, Katz CL, Reissman DB, Ozbay F, et al. The burden of full and subsyndromal posttraumatic stress disorder among police involved in the World Trade Center rescue and recovery effort. J Psychiatr Res. 2012;46(7):835-42.
84. Cann A, Calhoun LG, Tedeschi RG, Taku K, Vishnevsky T, Triplett KN, et al. A short form of the Posttraumatic Growth Inventory. Anxiety Stress Coping. 2010;23(2):127-37.

85. Blevins CA, Weathers FW, Davis MT, Witte TK, Domino JL. The Posttraumatic Stress Disorder Checklist for DSM-5 (PCL-5): Development and Initial Psychometric Evaluation. Journal of traumatic stress. 2015;28(6):489-98.
86. McCreary DR, Thompson MM. Development of two reliable and valid measures of stressors in policing: The operational and organizational police stress questionnaires. International Journal of Stress Management. 2006;13(4):494-518.
87. Carleton RN, Collimore KC, Asmundson GJG, McCabe RE, Rowa K, Antony MM. Refining and validating the Social Interaction Anxiety Scale and the Social Phobia Scale. Depress Anxiety. 2009;26(2):E71-81.
88. Mattick RP, Clarke JC. Development and validation of measures of social phobia scrutiny fear and social interaction anxiety. Behav Res Ther. 1998;36(4):455-70.
89. Reilly AR, Carleton RN, Weeks JW. Psychometric evaluation of the Social Interaction Phobia Scale. Anxiety, stress, and coping. 2012;25(5):529-42.
90. Menatti AR, Weeks JW, Carleton RN, Morrison AS, Heimberg RG, Hope DA, et al. The Social Interaction Phobia Scale: Continued support for the psychometric validity of the SIPS using clinical and non-clinical samples. J Anxiety Disord. 2015;32:46-55.
91. Weeks JW, Carleton RN, Asmundson GJG, McCabe RE, Antony MM. "Social Anxiety Disorder Carved at its Joints": Evidence for the taxonicity of social anxiety disorder. J Anxiety Disord. 2010;24(7):734-42.
92. Caron J. Une validation de la forme abrégée de l’Échelle de provisions sociales: l’ÉPS-10 items. Sante Ment Que. 2013;38(1):297-318.
93. Cutrona CE, Russell DW. The provisions of social relationships and adaptation to stress. Advances in personal relationships. 1987;1(1):37-67.
94. Chadwick P, Hember M, Symes J, Peters E, Kuipers E, Dagnan D. Responding mindfully to unpleasant thoughts and images: reliability and validity of the Southampton mindfulness questionnaire (SMQ). British Journal of Clinical Psychology. 2008;47(Pt 4):451-5.
95. Global Adult Tobacco Survey Collaborative Group. Global Adult Tobacco Survey (GATS): Core Questionnaire with Optional Questions. In: Prevention CfDCa, editor. Atlanta, GA: Centers for Disease Control and Prevention; 2020.
96. Sauer-Zavala S, Cassiello-Robbins C, Ametaj AA, Wilner JW, Pagan DS. Transdiagnostic Treatment Personalization: Prioritizing Unified Protocol treatment skills to capitalize on strengths or compensate for weaknesses. Behav Modif. 2019;43:518-43.
97. Sauer-Zavala S, Cassiello-Robbins C, Conklin LR, Bullis JR, Thompson-Hollands J, Kennedy KA. Isolating the Unique Effects of the Unified Protocol Treatment Modules Using Single Case Experimental Design. Behav Modif. 2017;41(2):286-307.
98. Sauer-Zavala S, Ametaj AA, Wilner JG, Bentley KH, Marquez S, Patrick KA, et al. Evaluating transdiagnostic, evidence-based mental health care in a safety-net setting serving homeless individuals. Psychotherapy (Chic). 2019;56(1):100-14.
99. Schaufeli W, Bakker AB, Salanova M. The Measurement of Work Engagement With a Short Questionnaire: A Cross-National Study. Educational and Psychological Measurement. 2006;66:701-16.
100. Seppala P, Mauno S, Feldt T, Hakanen JJ, Kinnunen U, Tolvanen A, et al. The construct validity of the Utrecht Work Engagement Scale: Multisample and longitudinal evidence. Journal of Happiness Studies. 2009;10(4):177-85.
